# Hypoxia-induced GBE1 expression promotes tumor progression through metabolic reprogramming in lung adenocarcinoma

**DOI:** 10.1038/s41392-020-0152-8

**Published:** 2020-05-22

**Authors:** Lifeng Li, Li Yang, Zhirui Fan, Wenhua Xue, Zhibo Shen, Yongliang Yuan, Xiangdong Sun, Dan Wang, Jingyao Lian, Liping Wang, Jie Zhao, Yi Zhang

**Affiliations:** 1 Biotherapy Center, The First Affiliated Hospital of Zhengzhou University, 450052 Zhengzhou, Henan China; 2 Cancer Center, The First Affiliated Hospital of Zhengzhou University, 450052 Zhengzhou, Henan China; 3 Internet Medical and System Applications of National Engineering Laboratory, Zhengzhou, China; 4 Integrated Traditional and Western Medicine, The First Affiliated Hospital of Zhengzhou University, 450052 Zhengzhou, Henan China; 5 Department of Pharmacy, The First Affiliated Hospital of Zhengzhou University, 450052 Zhengzhou, Henan China; 6 Marshall B.J. Medical Research Centre, Zhengzhou University, 450052 Zhengzhou, Henan China

**Keywords:** Cancer metabolism, Cancer metabolism

## Abstract

Hypoxia mediates a metabolic switch from oxidative phosphorylation to glycolysis and increases glycogen synthesis. We previously found that glycogen branching enzyme (GBE1) is downstream of the hypoxia-inducible factor-1 (HIF1) signaling pathway in lung adenocarcinoma (LUAD) cells; however, the molecular mechanism underlying HIF1 regulation of GBE1 expression remains unknown. Herein, the effect of GBE1 on tumor progression via changes in metabolic signaling under hypoxia in vitro and in vivo was evaluated, and GBE1-related genes from human specimens and data sets were analyzed. Hypoxia induced GBE1 upregulation in LUAD cells. GBE1-knockdown A549 cells showed impaired cell proliferation, clone formation, cell migration and invasion, angiogenesis, tumor growth, and metastasis. GBE1 mediated the metabolic reprogramming of LUAD cells. The expression of gluconeogenesis pathway molecules, especially fructose-1,6-bisphosphatase (FBP1), was markedly higher in shGBE1 A549 cells than it was in the control cells. FBP1 inhibited the tumor progression of LUAD. GBE1-mediated FBP1 suppression via promoter methylation enhanced HIF1*α* levels through NF-κB signaling. GBE1 may be a negative prognostic biomarker for LUAD patients. Altogether, hypoxia-induced HIF1*α* mediated GBE1 upregulation, suppressing FBP1 expression by promoter methylation via NF-κB signaling in LUAD cells. FBP1 blockade upregulated HIF1*α*, triggered the switch to anaerobic glycolysis, and enhanced glucose uptake. Therefore, targeting HIF1*α*/GBE1/NF-κB/FBP1 signaling may be a potential therapeutic strategy for LUAD.

## Introduction

Tumor hypoxia has been identified as a prognostic factor for poor patient outcomes^1,2^. It is likely that hypoxia induces oncogenes and other drivers of tumor progression^3^ to confer an aggressive phenotype^4^. Hypoxia induces a metabolic switch from oxidative phosphorylation to glycolysis and increased glycogen synthesis; this metabolic reprogramming is advantageous for tumor growth^5^. Although the mechanism of glycolysis regulation under hypoxia has been elucidated, the effect of hypoxia on glycogen metabolic fate needs to be addressed.

In our previous work, glycogen branching enzyme (GBE1) was shown to be downstream of the hypoxia-inducible factor-1 (HIF1) signaling pathway in lung cancer cells under hypoxia^6^. GBE1 is essential for the globular and branched structure of glycogen, increasing solubility by creating a hydrophilic surface and reducing intracellular osmotic pressure^7,8^.

It has been shown that hypoxia promotes glycogen accumulation in cells through HIF1*α* stabilization^9–12^. Notably, GBE1 levels were significantly increased under hypoxic conditions^12^, and GBE1 expression was significantly upregulated in U87MG xenografts treated with bevacizumab^13^. These findings indicate that GBE1 may have also been regulated via hypoxia-induced HIF signaling in the tumor microenvironment.

To our knowledge, we are the first to report that blocking GBE1 promotes the production of CCL5 and CXCL10, which also recruits CD8^+^ T lymphocytes into the tumor microenvironment, and GBE1 might be a potential target for achieving tumor regression in lung adenocarcinoma (LUAD)^14^. However, the importance and regulation of GBE1 in cancer biology and clinical oncology are unclear. In this study, the expression of GBE1 was significantly increased in hypoxia-conditioned primary LUAD cells and was highly positively associated with HIF1*α* expression. LUAD patients with high GBE1 expression exhibited worse survival than did lung squamous carcinoma patients, as evidenced by the analysis and integration of multiple data sets^6^. Herein, we demonstrate that GBE1 is an important transcriptional target of HIF1*α* signaling and can promote tumor progression by regulating the methylation of FBP1 via the NF-κB signaling pathway in LUAD cells.

## Results

### Hypoxia elevates GBE1 levels and glycogen production in LUAD cells

**Fig. 1:**
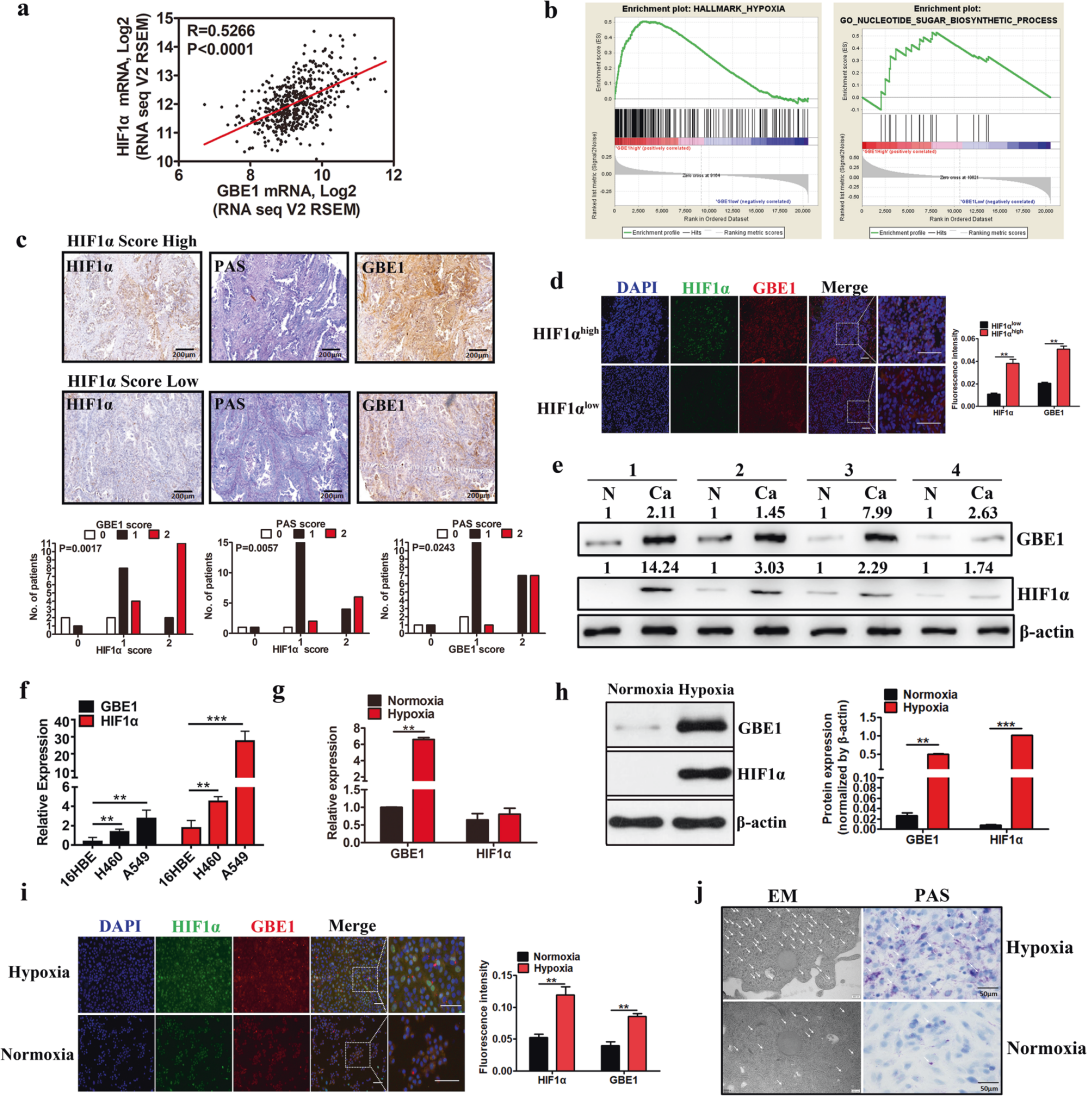
Hypoxia elevates GBE1 levels and glycogen production in LUAD cells. **a** Scatter plots showing the correlation between HIF1*α* and GBE1 expression. The red line represents the linear interpolation curve between both genes in the samples from LUAD patients. The correlation coefficient *R* value between two genes was computed using Pearson’s coefficient correlation. **b** Gene set enrichment analysis of The Cancer Genome Atlas (TCGA) data set revealed that GBE1 expression was significantly correlated with hallmark hypoxia and the nucleotide sugar biosynthetic process pathway. **c** Immunohistochemistry (IHC) staining of primary LUAD samples with high or low HIF1*α* and GBE1 expression scores and PAS staining for glycogen. **d** Immunofluorescence images of LUAD tissues stained for DNA (DAPI), HIF1*α* (green), and GBE1 (red) were merged. The scale bar represents 20 μm. **e** Protein expression of GBE1 and HIF1*α* in the LUAD and adjacent tissues was analyzed by western blotting. **f** mRNA expression of GBE1 and HIF1*α* in normal lung (16HBE) and cancer (H460 and A549) cell lines was analyzed by qPCR. **g** mRNA expression of *GBE1* and *HIF1α* in A549 cells under hypoxia or normoxia was analyzed by qPCR. **h** Protein expression of GBE1 and HIF1*α* in the A549 cells under hypoxia or normoxia was analyzed by western blotting. **i** Immunofluorescence images of the A549 cells under hypoxia and normoxia stained for DNA (DAPI), HIF1*α* (green), and GBE1 (red) were merged. Scale bar represents 20 μm. **j** Transmission electron microscopy and PAS staining of glycogen under hypoxia and normoxia. Data are represented as the means ± SD. **P < 0.01, ***P < 0.001

Hypoxia in the tumor microenvironment induces increased resistance to tumor therapy, including radiotherapy, chemotherapy, and immunotherapy^15–17^. ^18^F-fluoromisonidazole (^18^FMISO) positron emission tomography (PET) is used to investigate the magnitude and spatial distribution of tumor hypoxia. We found that tumor hypoxia and increased glucose intake were concurrent in stage III and IV LUAD patients (Supplementary Fig. S1a). The results of the tissue microarray including 30 LUAD samples showed that the expression of the hypoxia-relevant molecules HIF1*α* and vascular endothelial growth factor (VEGF) was significantly higher in the tumor tissues than it was in the peritumor tissues (Supplementary Fig. S1b). The gene expression profiling analysis based on the GSE30979 data set revealed that there was a significant alteration in molecules associated with HIF1, glycolysis/gluconeogenesis pathways, and metabolism enzymes (e.g., GBE1) in hypoxia-conditioned LUAD cells (Supplementary Fig. S1c). We next analyzed the correlation between HIF1*α* and GBE1 using The Cancer Genome Atlas (TCGA) data set and found that the GBE1 expression pattern was highly and positively correlated with HIF1*α* in LUAD (Fig. 1a). To further confirm whether GBE1 levels are associated with the metabolic pathway in LUAD cells, gene set enrichment analysis (GSEA) was performed^18^. Predefined gene sets involved in the metabolic pathway were remarkably enriched in the LUAD samples with a high level of GBE1 in the TCGA data set. The GSEA results indicated that “hallmark hypoxia” and “nucleotide sugar biosynthetic process” pathways had a significant effect on LUAD samples with high levels of GBE1 (Fig. 1b). Tissue microarray results revealed that tissues with a high score for HIF1*α* showed increased GBE1 expression as well as periodic acid-Schiff (PAS) staining, a major determinant of glycogen accumulation^13^, in hypoxic areas (Fig. 1c). Supporting the above findings, we found that HIF1*α* expression was mostly colocalized with GBE1 expression in primary LUAD samples, as determined by immunofluorescence assays (Fig. 1d). Moreover, GBE1 protein levels and HIF1*α* expression were obviously higher in tumor tissues than they were in the paired peritumor tissues (Fig. 1e).

In addition, the mRNA expression of GBE1 and HIF1*α* was also significantly higher in LUAD cells (A549) than it was in normal lung cells (16HBE; Fig. 1f). Furthermore, upon A549 cell exposure to hypoxia, the protein levels of HIF1*α* and GBE1 were markedly increased (Fig. 1h, i), and the mRNA level of GBE1 was also increased (Fig. 1g). Hypoxia-mediated GBE1 expression induced a more pronounced accumulation of glycogen in the LUAD cells, as evidenced by PAS staining and transmission electron microscopy evidence (Fig. 1j). To further confirm the effect of HIF1*α*/GBE1 on glycogen production, siHIF1*α* or siGBE1 A549 cells were generated. Knocking down HIF1*α* or GBE1 decreased the glycogen accumulation in the A549 cells (Supplementary Fig. S2a, b). Therefore, these findings indicated that hypoxia elevates GBE1 expression levels, further inducing glycogen accumulation in LUAD.

Next, we evaluated the relationship between GBE1 expression and clinical pathological parameters in the TCGA data set and found that the expression of GBE1 in the tumor tissues of late-stage LUAD patients was significantly increased (Supplementary Fig. S3a). Moreover, the TCGA data set analysis results demonstrated that GBE1 expression was closely associated with LUAD progression-related markers, including mutation of tumor protein p53 (TP53) and epidermal growth factor receptor (EGFR) (Supplementary Fig. S3b). The overall survival (OS) analysis indicated that LUAD patients with high GBE1 levels exhibited poor survival (Supplementary Fig. S3c). Taken together, these data reveal that GBE1 is a potential prognostic biomarker for LUAD in patients.

### GBE1 is a direct target gene of HIF1*α*

**Fig. 2:**
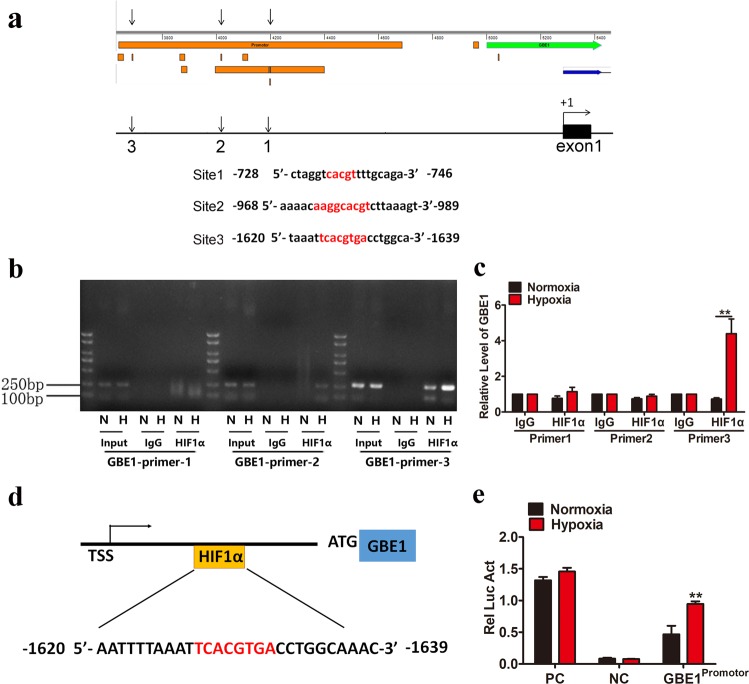
GBE1 is a direct target gene of HIF1α. **a** The PROMO and JASPAR websites predicted the possible binding sequences of *HIF1α* to the GBE1 promoter region. **b** A549 cells were exposed to hypoxia and normoxia for 24 h, and ChIP assays were performed using IgG or antibodies against HIF1*α*. Primers flanking the entire sequence are shown and used for qPCR; the results were normalized to those for IgG at normoxic O_2_. **c** HIF1*α* primers were used to detect the relative expression levels of *GBE1* by qPCR. **d** Schematic representation of the GBE1 promoter region. HIF1*α* in the yellow box indicates the location of the primer on the GBE1 promoter. **e** A549 cells were cotransfected with pSV-*Renilla* and firefly luciferase reporter pGL2-HRE1 (containing an oligonucleotide encompassing HIF-binding sites) and exposed to hypoxic and normoxic O_2_ for 24 h. Data are represented as the means ± SD. **P < 0.01

To determine whether HIF1*α* directly regulates GBE1 gene transcription, we performed ChIP assays. A549 cells were cultured under normoxia and hypoxia, and chromatin complexes were immunoprecipitated with an antihuman HIF1*α* antibody. PCR analysis was performed using specific primers for the human GBE1 promoter region, which encompasses identified hypoxia response element (HRE) sites. A DNA sequence, encompassing 5′-CACGT-3′ at −728 bp (site 1), 5′-AAGGCACGT-3′ at −968 bp (site 2), and 5′-TCACGTGA-3′ at −1620 bp (site 3) relative to the GBE1 transcription start site (Fig. 2a), was enriched in the fraction obtained by anti-HIF1*α* immunoprecipitation of the chromatin from A549 cells under hypoxia, indicating that the sequence between −1620 bp and −1639 bp in the GBE1 promoter region (site 3) is critical and preferential for HIF1*α* binding (Fig. 2b–d). The GBE1 promoter region exhibited an increased enrichment of HIF1*α* in the A549 cells under hypoxia, revealing that HIF1*α* is indeed located in the promoter region of GBE1 in hypoxic A549 cells (Fig. 2e). Accordingly, our findings, GBE1 is a transcriptional target of HIF1*α* in LUAD cells under hypoxia.

### GBE1 is a critical determinant of tumor progression

**Fig. 3:**
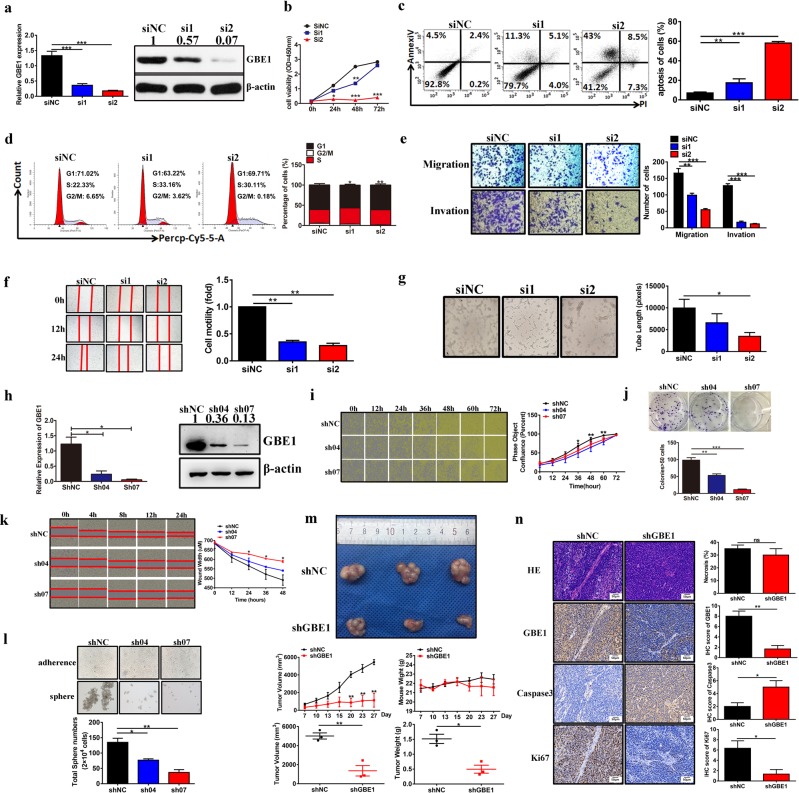
The effect of GBE1 on the biological behavior of LUAD in vitro and in vivo. **a** qPCR and western blot analysis confirming the effects of knocking down GBE1 in the siGBE1 A549 cells compared with those of the negative control cells. **a**–**g** siGBE1 A549 cell proliferation was analyzed by CCK-8 assay (**b**), apoptosis by flow cytometry (**c**), cell cycle by flow cytometry (**d**), migration and invasion by Transwell assays (**e**), migration by wound-healing assays (**f**), and angiogenesis by tube formation assays (**g**). **h** qPCR and western blot analysis confirming the knockdown of GBE1 in the shGBE1 A549 cells compared with the levels in the negative control cells. **i** Estimating the cell proliferation rate in shGBE1 cells was performed by IncuCyte ZOOM™ assay. **j**, **k** shGBE1 A549 cell colony formation (**j**), migration (**k**), and sphere formation ability (**l**). **m** Representative macroscopic tumor images upon necropsy of mice with postimplant shGBE1 and shNC A549 cells. Tumor volumes and body weights were measured at the indicated time points in the tumor-implanted mice after cell implantation. Tumor volumes and weights of xenografts at the final time point after cell implantation were also measured. **n** Representative IHC imaging of GBE1, caspase-3, and Ki67 expression and HE staining of tissues from xenografts in formalin-fixed paraffin-embedded sections. Data are represented as the means ± SD. *P < 0.05, **P < 0.01, ***P < 0.001

To analyze the effect of GBE1 on tumor behavior, transient GBE1-knockdown A549 cells (siGBE1 cells) were constructed; GBE1 knockdown efficacy in the A549 cells was analyzed by qPCR and western blotting (Fig. 3a). siGBE1 cell viability was obviously decreased compared with that of the control (Fig. 3b), while cell apoptosis was markedly enhanced, according to the flow cytometry results (Fig. 3c). The cell cycle profile analysis of the siGBE1 cells showed a decreased percentage of cells in the G2/M phase with a concomitant increase in the percentage of cells in the S/G1 phase compared with these percentages of the controls (Fig. 3d). Moreover, the Transwell, wound-healing, and tube formation assays showed that siGBE1 cells had decreased cell migration and invasion ability, as well as impaired angiogenesis, compared with these indicators in the control cells (Fig. 3e–g).

To further evaluate the effect of GBE1 on tumor progression, stable GBE1-knockdown A549 cells (shGBE1 cells) were generated (Fig. 3h). After GBE1 knockdown, the cell proliferation and colony formation ability were markedly decreased (Fig. 3i, j), while the cell apoptosis rate was increased (Supplementary Fig. S4a). The cell cycle profiling results (Supplementary Fig. S4b) and the decreased cell migration ability (Fig. 3k) were similar to those of the siGBE1 cells. Moreover, the sphere-forming ability of the shGBE1 cells was significantly reduced compared with that of the control (Fig. 3l). In addition, GBE1 knockdown in the A549 cells increased their sensitivity to radiotherapy and chemotherapy with docetaxel (Supplementary Fig. S4c–e). Next, we chose to use GBE1-sh07 for further experiments in vivo. In a xenograft experiment, shGBE1 A549 cells or shRNA scramble A549 cells were subcutaneously injected into nude mice. Notably, blocking GBE1 resulted in a marked reduction in tumor growth, as indicated by tumor volume and weight, compared with the volume and weight of the control (Fig. 3m). Furthermore, we found that GBE1 and Ki67 expression in the xenografts with blocked GBE1 was significantly decreased compared with that of the control; the opposite pattern was found for caspase-3 expression (Fig. 3n). Last, A549 cell metastasis to the lung was evaluated, and the number of metastatic lesions after blocking GBE1 was dramatically decreased compared with the number in the control (Supplementary Fig. S4f, g). In addition, since GBE1 is upregulated under hypoxia, some tests were also performed under hypoxia to detect a role for GBE1 in the hypoxia response. The results showed that GBE1 promoted tumor progression under hypoxia in vitro (Supplementary Fig. S5). Collectively, GBE1 is a critical determinant of tumor progression and represents a potential therapeutic target for tumor treatment.

### GBE1 induces the metabolic reprogramming of LUAD cells

**Fig. 4:**
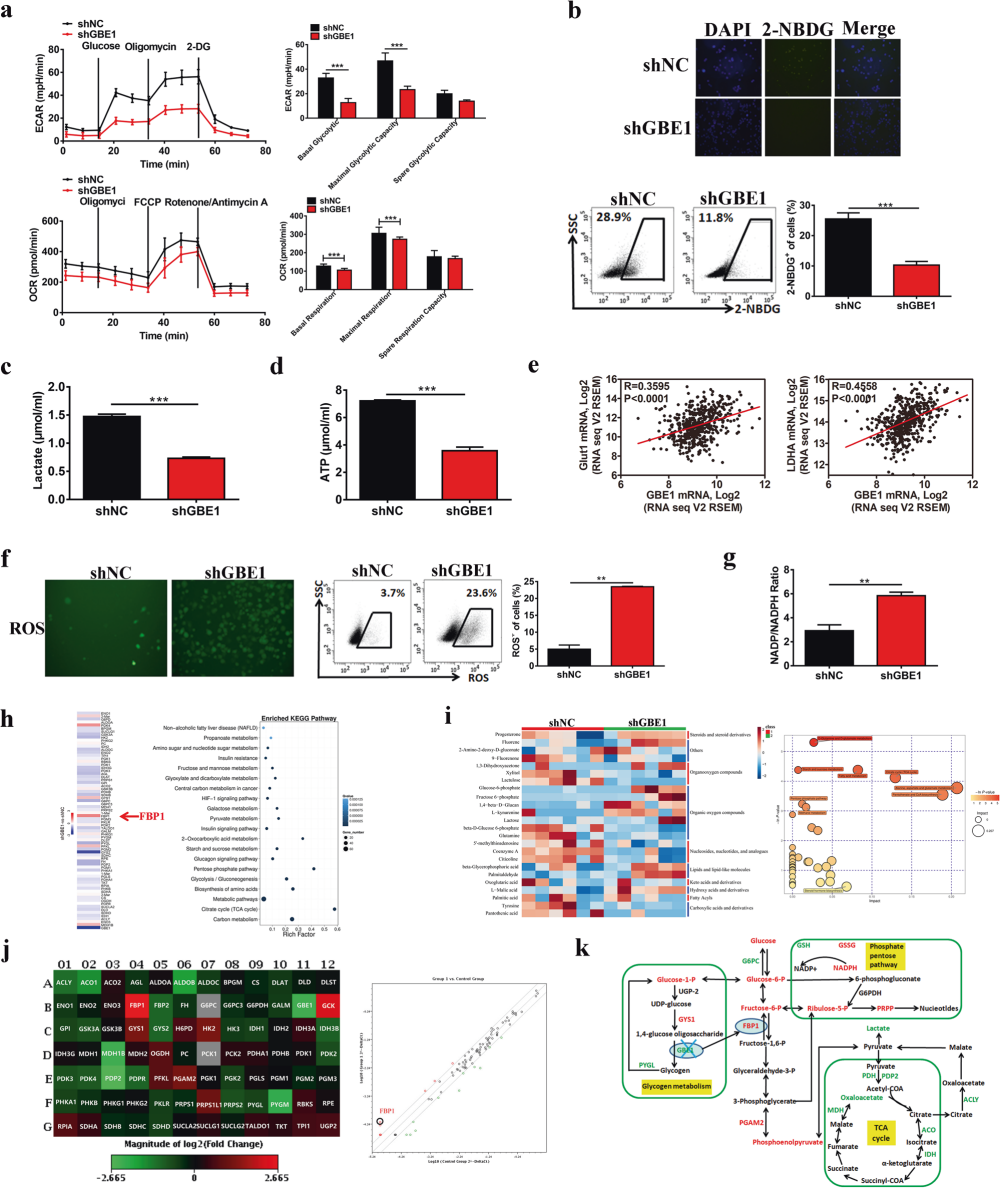
GBE1 induces metabolic reprogramming in LUAD cells. **a** Seahorse metabolic analysis of ECARs and OCRs in the shGBE1 and shNC A549 cells. **b** Intracellular 2-NBDG accumulation evaluation in the shGBE1 and shNC A549 cells. **c**, **d** The effect of GBE1 knockdown in the A549 cells on lactate production (**c**) and ATP secretion (**d**). **e** The correlation between GBE1 expression levels in the LUAD tissues with Glut1 and LDHA expression levels in the samples in the TCGA data set. **f** ROS expression in the shGBE1 and shNC A549 cells was analyzed by fluorescence imaging and flow cytometry. **g** The ratio of NADP/NADPH in the shGBE1 and shNC A549 cells. **h** RNA-seq and **i** pan-metabolomic analysis of GBE1 knockdown in the A549 cells. **j** Heat map showing the fold changes of differentially expressed genes based on the glucose metabolism PCR array. **k** Schematic illustration of changes in metabolic signaling pathways induced by GBE1 knockdown. Data are represented as the means ± SD. **P < 0.01, ***P < 0.001

To further assess the effect of GBE1 on the bioenergetic profiling of LUAD, we analyzed the extracellular acidification rate (ECAR) and oxygen consumption rate (OCR) of GBE1-knockdown A549 cells. Knocking down GBE1 led to a decrease in the ECAR and OCR parameters (Fig. 4a). Moreover, GBE1 knockdown decreased the metabolic transition from glycolysis to mitochondrial oxidative phosphorylation (Fig. 4a). To evaluate the function of GBE1 in glycolytic metabolism, we used the fluorescent glucose analog 2-NBDG^19^ and found a decreased uptake of glucose in the shGBE1 cells compared with that of the control (Fig. 4b). In addition, we analyzed lactate and ATP levels using the relevant kits and found that lactate and ATP production was markedly decreased in the shGBE1 cells compared with that of the control (Fig. 4c, d). Moreover, GBE1 levels were closely associated with the Glut1 and LDHA levels in the TCGA data set (Fig. 4e). These findings indicate that glycolysis was impaired in the LUAD cells after GBE1 knockdown.

Intracellular reactive oxygen species (ROS) were significantly higher in the A549 shGBE1 cells than they were in the control cells (Fig. 4f). Because the GBE1 blockade was correlated with ROS increase and inhibition of cell proliferation, we theorized that the pentose phosphate pathway (PPP) was affected by the lack of glycogen metabolism in the absence of GBE1. Moreover, NADPH, as a reducing agent, plays an important role in nucleotide, amino acid and lipid synthesis as well as ROS scavenging. The results demonstrated a significant increase in the NADP^+^/NADPH ratio in the shGBE1 cells (Fig. 4g). Through RNA-sequencing (RNA-seq) and pan-metabolomic GC/LC-MS analysis, the expression of metabolic pathway-related genes, especially gluconeogenesis pathway molecules (e.g., FBP1), was found to be markedly higher in the shGBE1 cells than it was in the control cells (Fig. 4h, i). All expressed genes in the shGBE1 and control cells are presented in a scatter plot and volcano graph (Supplementary Fig. S6a, b). A pathway analysis based on gene ontology (GO) and KEGG data sets was also performed (Supplementary Fig. S6c, d). To determine the downstream metabolic targets of GBE1, a glucose metabolism PCR array was used to evaluate changes in metabolic genes after GBE1 knockdown, with the results showing that 11 of 84 genes were downregulated by ≥2-fold. Conversely, nine genes were found to be upregulated by ˃2-fold in the shGBE1 cells, with FBP1 exhibiting the greatest upregulation (6.255-fold; Fig. 4j and Supplementary Fig. S6e). Taken together, the results indicate that high GBE1 levels led to a dominant role of glycogen metabolism in LUAD, whereas blocking GBE1 induced FBP1 expression, which is critical for the glycolysis and PPP pathways (Fig. 4k).

### FBP1 prevents LUAD tumor progression

**Fig. 5:**
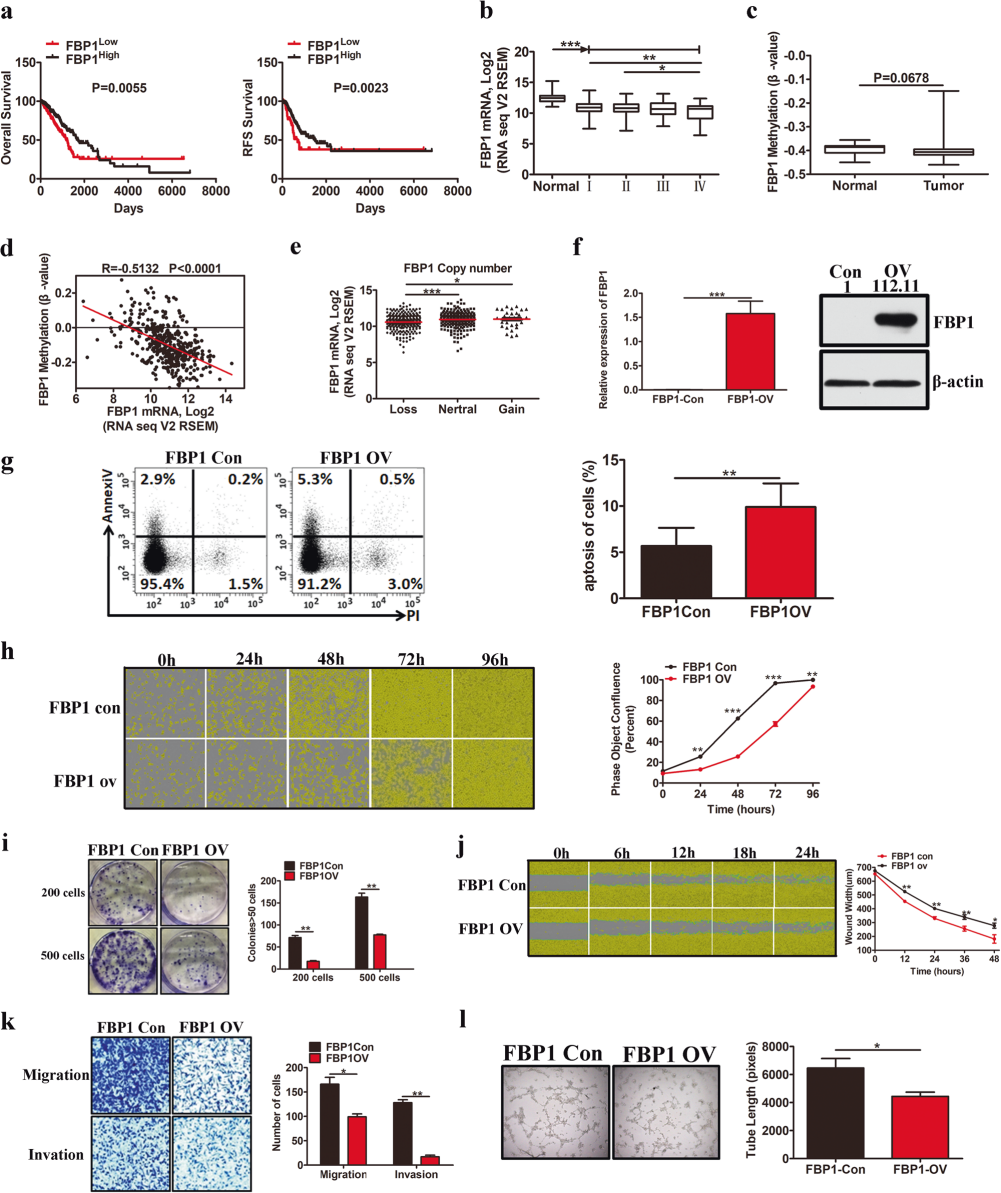
FBP1 prevents LUAD tumor progression. **a** Kaplan−Meier OS and RFS curves based on FBP1 expression as determined using the TCGA data set. **b** Box plots of FBP1 expression in the LUAD tissues at different tumor stages, according to the TCGA data set. **c** Scatter plot of FBP1 methylation levels in the tumor and adjacent tissues from samples in the TCGA data set. **d** The correlation between FBP1 mRNA levels in the LUAD tumor samples and FBP1 methylation levels in the TCGA data set. **e** Scatter plot of FBP1 expression was based on FBP1 copy number alterations in the TCGA data set. **f** Western blotting and qPCR analysis confirming the overexpression of FBP1 in the A549 cells compared with the level in the control cells. **g**–**l** The effect of FBP1 overexpression on cell apoptosis (**g**); cell proliferation (**h**); colony formation (**i**); cell migration, as determined by wound-healing assays (**j**); cell migration and invasion, as determined by Transwell assays (**k**); and angiogenesis (**l**). Data are represented as the means ± SD. *P < 0.05, **P < 0.01, ***P < 0.001

FBP1, which can reduce the levels of fructose-1,6-biphosphate within cells, is silenced in many tumors^20^. To investigate the effect of FBP1 on LUAD tumor progression, we first analyzed the correlation between FBP1 level and survival using the TCGA data set. The results showed that LUAD patients with low FBP1 levels had poor OS and relapse-free survival (RFS; Fig. 5a). Moreover, FBP1 expression was markedly lower in the tumor tissues than it was in the normal lung tissues (Fig. 5b) and was markedly lower in late stage (IV) LUAD tissues than it was in early stage (I, II) LUAD tissues (Fig. 5b). These data indicate that the absence of FBP1 expression is correlated with LUAD tumor progression.

To further investigate the mechanism behind the suppression of FBP1 expression and LUAD tumor progression, we analyzed the promoter methylation and copy number profiling of FBP1 using the TCGA data set and found that FBP1 promoter methylation was likely to be higher in tumor tissues than in normal lung tissues (Fig. 5c). Moreover, the FBP1 level was inversely associated with the degree of FBP1 methylation in the LUAD tissues (Fig. 5d), suggesting that methylation of the FBP1 promoter inhibited FBP1 transcription in LUAD cells. In addition, the loss of FBP1 copy number was closely related to decreased FBP1 expression (Fig. 5e).

Next, we successfully established stable FBP1 overexpression in A549 cells (Fig. 5f) to investigate the effect of FBP1 on LUAD tumor progression. Consistent with previous studies in ccRCC^21^, HCC^22^, and breast cancer cells^23^, stable overexpression of FBP1 increased A549 cell apoptosis (Fig. 5g) and inhibited A549 cell proliferation (Fig. 5h) and colony formation ability (Fig. 5i). Furthermore, cell migration, invasion, and angiogenesis abilities were markedly decreased after FBP1 overexpression (Fig. 5j–l). These findings indicate that LUAD exhibits low levels of FBP1 and that FBP1 overexpression can attenuate LUAD tumor progression.

### GBE1-mediated FBP1 suppression via promoter methylation enhances HIF1*α* levels through NF-κB signaling

**Fig. 6:**
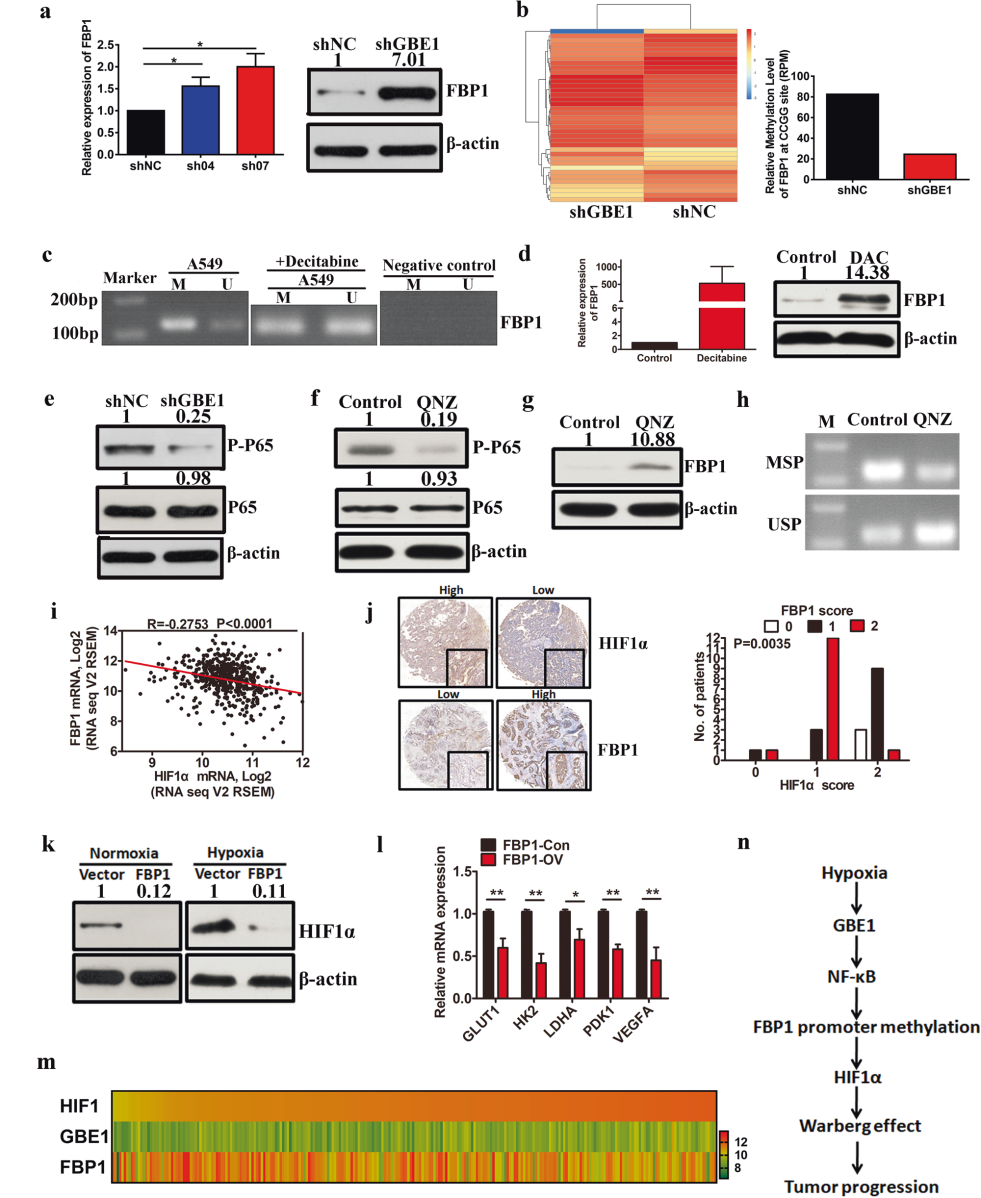
GBE1-mediated FBP1 suppression via promoter methylation enhances HIF1α levels through NF-κB signaling. **a** qPCR and western blotting were performed to examine the mRNA and protein levels of FBP1 in the shGBE1 A549 cells. **b** FBP1 methylation levels in the shGBE1 and shNC A549 cells were analyzed by MethylRAD technology. **c** MSP analysis of the FBP1 promoter in the A549 cells treated with or without decitabine (5 μmol L^−1^, treated for 6 d). M methylated-specific primers, U unmethylated specific primers. **d** FBP1 expression was detected by qPCR and western blotting in the A549 cells after treatment with decitabine. **e** Protein levels of phospho-p65 (p-p65) and total p65 (p65) in the shGBE1 and shNC A549 cells were analyzed by western blotting. **f** Protein levels of phospho-p65 and total P65 in the A549 cells treated with QNZ were analyzed by western blotting. **g** Protein levels of FBP1 in the A549 cells treated with QNZ were analyzed by western blotting. **h** MSP analysis of the FBP1 promoter in the A549 cells treated with QNZ. **i** Correlation between FBP1 expression levels in the LUAD samples and HIF1*α* expression in the TCGA data set. **j** IHC of the LUAD tissues with high or low HIF1*α* and FBP1 expression. **k** HIF1*α* expression in the A549 cells with or without FBP1 overexpression under normoxia and hypoxia (1% O_2_) following 24 h incubation was analyzed by western blotting. **l** mRNA levels of *GLUT1*, *HK*2, *LDHA, PDK1*, and *VEGFA* in the A549 cells with or without FBP1 overexpression were analyzed by qPCR. **m** Heat map showing the expression of HIF1*α*, GBE1, and FBP1 in the LUAD tissues in the TCGA data set. **n** The signaling pathway of hypoxia-induced tumor progression. Hypoxia drives the Warburg effect to promote tumor progression via GBE1 induction, and GBE1-mediated FBP1 suppression by FBP1 promoter methylation enhances HIF1*α* levels via NF-κB signaling in LUAD. Data are represented as the means ± SD. *P < 0.05, **P < 0.01

Although GBE1 was assessed as a key downstream target of the HIF1*α* signaling pathway, we did not uncover the mechanism of GBE1 regulation in LUAD. After GBE1 knockdown in the A549 cells, the mRNA and protein levels of FBP1 were upregulated (Fig. 6a). As NF-κB may function in the epigenetic downregulation of FBP1 in tumor cells^24^, we theorized that blocking GBE1 increased the FBP1 expression and was accompanied by a decrease in FBP1 promoter methylation, which was confirmed by whole-genome DNA methylation profiling (Fig. 6b). Indeed, one typical CpG island (CGI) was found near FBP1 exon 1 (Supplementary Fig. S7a). Bisulfite genomic sequencing (BSP) was performed on A549 cells to investigate the methylation status of the FBP1 CpG island. BSP showed that the FBP1 promoter is hypermethylated in the A549 cells (Supplementary Fig. S7b). Furthermore, we sequenced the CpG-rich region near the FBP1 transcription initiation site (spanning 63 CpG sites) (Supplementary Fig. S7c, d). To evaluate the methylation of the FBP1 CpG island, methylation-specific PCR (MSP) analysis was performed, which detects methylated alleles; the CpG island in FBP1 was found to be partly demethylated after treatment with the methyltransferase inhibitor decitabine (5 μM; Fig. 6c). Next, we treated the A549 cells with decitabine for 3 d and discovered that FBP1, the mRNA and protein levels were markedly increased (Fig. 6d).

In addition, we also evaluated whether the NF-κB signaling pathway is essential for the GBE1 regulation of FBP1 methylation. shGBE1 A549 cells exhibited lower phospho-P65 levels compared with those of the control (Fig. 6e). As expected, FBP1 expression was restored after treatment with QNZ (EVP4593), an inhibitor of NF-κB, which mediated the downregulation of phospho-P65 (Fig. 6f, g). Moreover, the FBP1 promoter was partially demethylated after treatment with QNZ (Fig. 6h), suggesting that FBP1 is silenced via promoter methylation in an NF-κB-dependent manner.

Furthermore, a significant negative relationship between FBP1 and HIF1*α* expression was determined based on the TCGA data set (Fig. 6i). The immunohistochemistry (IHC) results also demonstrated a negative correlation between HIF1*α* and FBP1 levels in the LUAD tissues (Fig. 6j). FBP1 overexpression in the A549 cells led to a reduction in HIF1*α* expression under normoxia, which was further reduced under hypoxia (Fig. 6k). We also analyzed the expression of HIF1*α* target genes, including *GLUT1, HK2, LDHA, PDK1*, and *VEGFA*, and found that they were downregulated in the FBP1-overexpressing A549 cells compared with their levels in the controls (Fig. 6l). Data obtained from the TCGA data set showed that, following the increase in HIF1*α* expression, GBE1 expression was also increased and that of FBP1 was decreased in the LUAD tissues (Fig. 6m). The TCGA data set analysis indicated that HIF1*α* expression may be closely associated with LUAD tumor progression (Supplementary Fig. S7e). Taken together, the results indicate that hypoxia supports the Warburg effect to promote tumor progression via GBE1 induction and that GBE1-mediated FBP1 suppression via FBP1 promoter methylation enhances HIF1*α* levels through NF-κB signaling in LUAD tissues (Fig. 6n).

### Flavopiridol as a potential inhibitor of GBE1 for LUAD treatment

Next, we used an in vitro GBE1 assay to identify flavopiridol as an inhibitor of GBE1 from a small molecule compound library (Supplementary Fig. S8a). Flavopiridol attenuated A549 cell proliferation in a dose-dependent manner (Supplementary Fig. S8b), whereas shGBE1 A549 cells were resistant to flavopiridol treatment. After treatment with flavopiridol, GBE1 expression in A549 cells was markedly decreased (Supplementary Fig. S8c, d), which led to decreased intracellular glycogen levels (Supplementary Fig. S8e). Treatment with flavopiridol for 24 h resulted in increased cell apoptosis and decreased clone formation, migration, and invasion ability (Supplementary Fig. S8f).

To determine whether flavopiridol is sufficient to inhibit tumor progression in vivo, we used a xenograft mouse model. After cell injection for 3 d, mice were randomly divided into two groups and injected with flavopiridol or dimethyl sulfoxide (DMSO) as a control for 41 d. Compared with the control, we found that tumor growth in flavopiridol-treated mice was inhibited and that tumor volume and weight were significantly lower (Supplementary Fig. S8g, h). After xenografts were collected for further detection of GBE1 expression by IHC and western blotting, we found that GBE1 expression in flavopiridol-treated mouse xenografts was dramatically decreased compared with that of the control (Supplementary Fig. S8i). We also observed that treatment with the selected dose (20 μM) of flavopiridol did not cause any noticeable damage to organs and tissues (Supplementary Fig. S8j) nor did it significantly affect the hepatic and renal properties of nude mice (Supplementary Fig. S8k), indicating that flavopiridol treatment at this dose has minimal toxicity in vivo. These findings demonstrate that flavopiridol inactivates GBE1 to inhibit the tumor progression of LUAD and that flavopiridol is a promising therapeutic drug for LUAD.

### GBE1 is a negative prognostic biomarker for LUAD patients

**Fig. 7:**
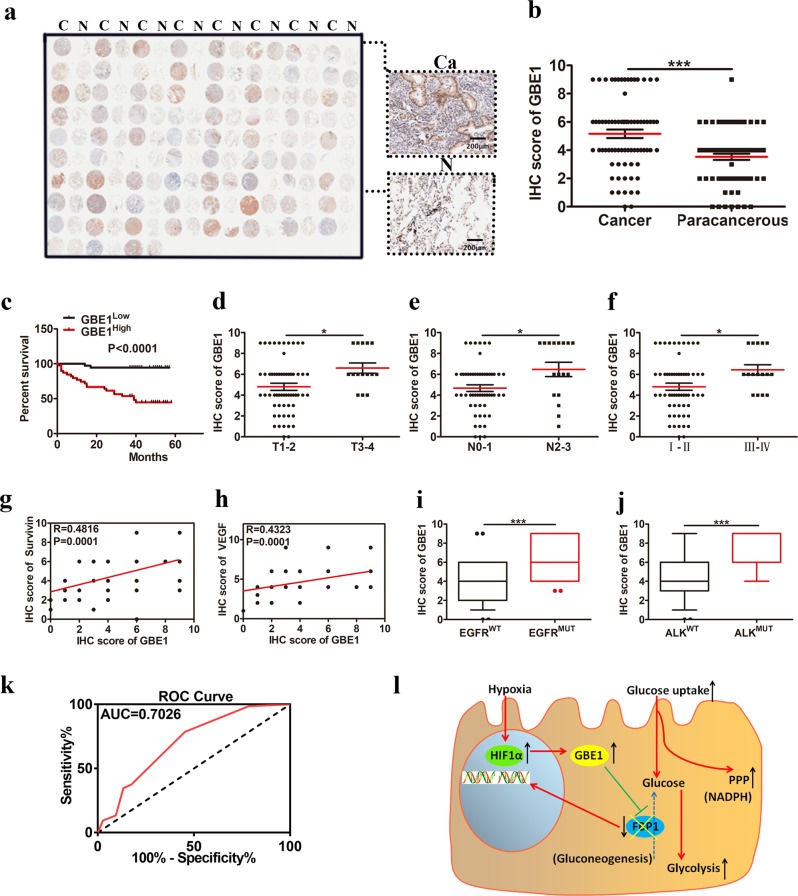
GBE1 is a negative prognostic biomarker for LUAD patients. **a** IHC staining of GBE1 expression from a representative human LUAD tissue microarray. C cancer tissue, N adjacent normal lung. **b** Quantification of the IHC staining showing GBE1 expression in 75 LUAD and paired normal lung tissues. **c** Kaplan−Meier OS curve based on high or low GBE1 expression. **d**–**f** Scatter plot of GBE1 expression based on IHC score in the LUAD tissues with primary tumors (**d**) in regional lymph nodes (**e**) and by tumor stage (**f**). **g**, **h** Correlation between survivin and VEGF expression with GBE1 in the LUAD tissues. **i**, **j** IHC score of GBE1 in the LUAD tissues according to EGFR or ALK mutation status. **k** ROC curve based on GBE1 expression in the LUAD tissues. **l** Graphical summary of the metabolic pathway of GBE1 under hypoxia in the LUAD cells. Data are represented as the means ± SD. *P < 0.05, ***P < 0.001

We next investigated whether GBE1 expression had prognostic value by using tumor tissues from a clinically annotated cohort of 75 LUAD patients (Fig. 7a). The data indicated that GBE1 protein expression was enhanced in the 75 LUAD tissues compared with that in the paired normal lung tissues (Fig. 7b). LUAD patients with high levels of GBE1 in tumor tissues had a worse OS (Fig. 7c and Supplementary Fig. S9c), suggesting that GBE1 is an independent prognostic marker of OS. We also found that GBE1 expression was progressively increased in LUAD tissues according to the TNM staging system based on tumor size (Fig. 7d), N staging (Fig. 7e) and tumor stage (Fig. 7f), with a similar but weak reactivity observed in the paired normal lung tissues (Supplementary Fig. S9d–f). GBE1 expression was also closely associated with the expression of other disease progression-related markers, including survivin (Fig. 7g and Supplementary Fig. S9a), VEGF (Fig. 7h and Supplementary Fig. S9b), mutated EGFR (Fig. 7i), and anaplastic lymphoma kinase (ALK; Fig. 7j), which was similar to the expression observed in the paired normal lung tissue (Supplementary Fig. S9g–j). Finally, consistent with this prognostic profiling, GBE1 expression was elevated—as determined by receiver operating characteristic (ROC) curve analysis, where the area under the curve (AUC) of GBE1 expression in the LUAD tissues in the TCGA data set was 67.7% (Fig. 7k)—indicating that GBE1 can be considered a prognostic biomarker for LUAD.

## Discussion

Hypoxia is one of the characteristics of the tumor microenvironment, where it increases tumor aggressiveness and exerts an adverse effect on patient prognosis. Under hypoxia, the growth and energy metabolism of normal cells is obviously damaged, whereas cancer cells adapt to maintain tumor growth through metabolic switching from oxidative phosphorylation to oxygen-independent glycolysis. By conferring such a growth advantage to tumor cells, the Warburg effect is considered a basal feature of tumor cells^25–27^. It has been well established that glycogen plays a key role in promoting cell survival under hypoxia in normal and cancer cells, and studies have also suggested that glucose transfer through glycogen may enhance the survival of tumor cells exposed to hypoxia^12,13,28–31^.

A previous study based on genomic data indicated that the levels of GBE1 in acute myelocytic leukemia^32^ and glycogen metabolism-related genes, including GBE1 and carbonic anhydrase IX (CA9), in bevacizumab-treated tumors^33^ were upregulated. Additionally, GBE1 levels are associated with the efficacy of anti-PD1 treatment in melanoma patients^31^. Similarly, in this study, we analyzed the microarray gene expression data (GSE30979) of ten patients with lung cancer via gene expression profiling analysis and biomedical gene information in the Cloud and found that GBE1 had a significant association with hypoxia. However, the mechanisms that drive GBE1 expression in lung cancer are still poorly understood.

Similar to GBE1, HIF1*α* is increased in late-stage tumors and is closely correlated with tumor progression^2^. HIF1*α*-mediated gene expression led to increased cellular oxygen delivery by angiogenic factor production or provided a survival advantage when cells were subjected to decreased oxygen availability^34^. There is a wealth of data for both the causes of the Warburg effect, which occurs predominantly through HIF activity, and the downstream effects of HIF activation that enable cellular adaptation to hypoxia. However, the hypoxia-mediated molecular mechanism of tumor progression via GBE1 is not fully understood. In this study, we found that hypoxia induced a significant increase in GBE1 levels in lung cancer cells. Moreover, GBE1 was also found in hypoxic areas of the xenografts. Using ChIP assays, we demonstrated that enhanced HIF1*α* was bound to the promoter of GBE1 after hypoxia exposure. This is the first study to show that GBE1 is transcriptionally regulated by HIF1*α* and that GBE1, a critical downstream effector of HIF1*α*, affects tumor progression. In addition, the clinical significance of this signaling pathway was confirmed by data from human LUAD samples, suggesting that the clinical impact of HIF1*α* expression is elevated via GBE1 coexpression. All these findings contribute to an answer explaining why GBE1 is such a powerful prognostic factor for LUAD.

Few studies have been performed using GBE1-knockout mice because of the occurrence of hydrops fetalis resulting from glycogen storage disease type IV^35^. GBE1 deficiency is correlated with an increase in insoluble polysaccharide particles, which induces autosomal recessive glycogen storage disorder type IV, a severe disorder with a variable age of onset^36^. In the current study, GBE1 depletion had a profound effect on malignant cells under hypoxia, suggesting that GBE1 expression protects cells from hypoxia and allows them to survive under this harsh condition to further promote local proliferate and metastasize. Moreover, this research showed that GBE1 expression was necessary for cancer progression.

Our RNA sequencing and metabolomic analysis revealed that the majority of changes driven by GBE1 knockdown included a series of metabolic pathways, such as glycogen metabolism, glycolysis/gluconeogenesis, tricarboxylic acid cycle, and PPP, and changes in the expression levels of the associated metabolic zymogram. Decreased GBE1 expression directly affected not only the production of glycogen but also glucose metabolic signaling pathways, ultimately inhibiting lung cancer cell growth. One dominant function of these pathways is meeting the energy required for cell proliferation^37^. At the same time, we observed that, in response to GBE1 knockdown, the production of gluconeogenesis-related metabolites, such as glucose-6-phosphate, fructose-6-phosphate, and glucose-1-phosphate, was increased. The expression of related metabolic enzymes, such as FBP1 and phosphoglycerate mutase 2 (PGAM2), was also upregulated. A comprehensive commercial PCR array and subsequent experimental verification demonstrated that GBE1 markedly regulates the expression and function of FBP1; this finding indicates that the mechanism by which GBE1 affects cell development involves more than simple regulation of energy metabolism and has important roles in the relevant metabolic molecular pathways within cells.

FBP1, a rate-limiting enzyme that decreases the levels of fructose-1,6-bisphosphate in gluconeogenesis, was downregulated via promoter methylation in an NF-κB-dependent manner^24^. FBP1 expression is decreased in multiple types of cancer^21,23,38,39^. FBP1 is regarded as a suppressor of tumors, and a decrease in FBP1 is positively correlated with the poor prognosis for people with carcinoma. Further studies showed that FBP1 suppresses tumor progression by inhibiting the Warburg effect^23^ and function of transcription factor HIF1*α*^21,40^. Moreover, methylation of the FBP1 promoter was analyzed and acts as an independent prognostic predictor for people with tumors. Importantly, NF-κB appears to be crucial for the methylation of the FBP1 promoter in tumor cells^24^. In the present study, we identified a novel function for FBP1 in inhibiting tumor progression and explored whether the abnormal regulation of FBP1 is involved in GBE1-induced cellular transformation and carcinogenesis. The importance and biological consequences of this interaction remain unclear.

LUAD metabolism is an important issue and an interesting target for therapy. In addition to glycolysis, the glycogen metabolism signaling pathway plays a key role in cancer progression. In particular, inhibiting glycogenesis may reduce tumor growth and can be combined with traditional therapy. It may be promising to explore specific drugs and target glycogenic flux, which depends on the glycogen metabolism signaling pathway. Suppression of GBE1 may also be an interesting strategy for use in supplemental therapy for malignancies because it can leverage the metabolic changes in transformed cells. Flavopiridol is a synthetic flavonoid that can inhibit many cyclin-dependent kinases (CDKs), including CDK1, 2, 4, 7, and 9^41^. Flavopiridol has also been shown to have an antitumor response by promoting the apoptosis of tumor cells and inhibiting angiogenesis^42,43^, and it has been reported to inactivate glycogen phosphorylase, which decreases glucose availability for glycolysis^44^. Other studies detected the effect of flavopiridol on glycolytic signaling pathway and observed that glucose metabolism-related enzymes, such as GLUT1, 3, and 4, hexokinase II, pyruvate kinase, and glycogen phosphorylase, which are known to be upregulated in glioblastoma cells, were significantly downregulated upon flavopiridol treatment^45^. In the present study, our data show that flavopiridol inhibits tumor progression by affecting the biological functions of tumors, such as decreasing cell proliferation, arresting the cell cycle, and increasing apoptosis, as observed in several in vitro and in vivo experiments. Moreover, flavopiridol treatment strongly decreased glycogen storage and GBE1 expression at the gene and protein levels in a dose-dependent manner. Thus, we provide a novel therapeutic perspective for flavopiridol use as a way to inhibit cell proliferation by showing that it induces a metabolic change that leads to cell death.

In summary, this study provides novel insight showing that hypoxia-induced HIF1*α* mediates GBE1 upregulation, which suppresses FBP1 expression by promoter methylation via NF-κB signaling in the tumor microenvironment of LUAD. Downregulation of FBP1 results in HIF1*α* enhancement, a switch to anaerobic glycolysis, and increased glucose uptake by LUAD cells (Fig. 7l). Therefore, therapeutic strategies that target the HIF1*α*/GBE1/NF-κB/FBP1 axis may represent an effective treatment for LUAD.

## Materials and methods

### Patients and samples

Tumor tissues for IHC, immunofluorescence, and western blotting were obtained from LUAD patients at our hospital. The patients were subjected to laboratory diagnosis by conventional cytology and signed informed consent forms. The consent procedure was in accordance with the standards from the Institutional Review Boards of our hospital.

### Public data set and GSEA

We obtained RNA-seq and clinical data of 571 LUAD cases from the TCGA data set. Methylation array, single nucleotide polymorphism array, and whole-exome sequencing data in the TCGA data set were also obtained to investigate the epigenetic and genomic regulation of FBP1.

To determine whether predefined gene sets based on glucose metabolism and prognosis in patients with LUAD showed a significant association with GBE1 expression in LUAD cases, a GSEA was performed^18^. The gene sets extracted from the Broad Institute database can be found under SHEDDEN_LUNG_CANCER_POOR_SURVIVAL_A6 (http://software.broadinstitute.org/gsea/msigdb/cards/SHEDDEN_LUNG_CANCER_POOR_SURVIVAL_A6.html).

### Cell culture and hypoxia

The normal lung epithelium cell line 16-HBE was cultured in Dulbecco’s Modified Eagle Medium (DMEM) (Gibco; Thermo Fisher Scientific, Waltham, MA, USA) with 10% fetal bovine serum (FBS) (HyClone; Thermo Fisher Scientific) in a 5% CO_2_ atmosphere at 37 °C. The A549 human lung cancer cell line was cultured in RMPI 1640 (HyClone) with 10% FBS (HyClone), penicillin (100 units mL^−1^), and streptomycin (100 μg mL^−1^) at 37 °C in a 5% CO_2_ humidified incubator. For hypoxic treatment, the cells were incubated in a hypoxia chamber with 1% O_2_ balanced with CO_2_ and nitrogen (Precision Scientific, USA).

### Plasmid construction and cell sorting

Protocols for the plasmid construction of small interfering RNA (siRNA) and short hairpin RNA (shRNA) against GBE1 (siGBE1 and shGBE1) were described in our previous study^14^. For FBP1 overexpression, a full-length cDNA insert of human FBP1 (1017 bp) was amplified by PCR using the following primers: forward, 5′-ATAAGAATGCGGCCGCGCCACCATGGCTGACCAGGCGCCCTTCGAC-3′ and reverse, 5′-TTCCTAGGTCACTGGGCAGAGTGCTTCTCATA-3′. The obtained sequences were subcloned into a pWSLV-07 vector (ViewSolid, Beijing, China) using 5′ *Not*I and 3′ *Avr*II restriction sites. To generate lentiviral particles, recombinant plasmids (GBE1-shRNA or FBP1-overexpression vectors) were transfected into 293T cells. The cells were infected by incubation with medium containing the lentiviral supernatant for 48 h. The cells were sorted by MoFlo XDP (Beckman Coulter, Brea, CA, USA) on the basis of the green fluorescent protein (GFP) expression.

### RNA isolation and quantitative real-time polymerase chain reaction (PCR)

Total RNA was extracted from lung cancer cells and tissues using TRIzol reagent (Invitrogen Life Technologies). Real-time quantitative PCR was performed in an Agilent Mx3005P using SYBR qPCR Mix (MQ10201s, Monad). The samples were amplified under the following conditions: 40 cycles of 95 °C/30 s, 95 °C/5 s, and 60 °C/30 s. The mRNA abundance of each gene of interest was normalized to that of β-actin. Data were analyzed by 2^−ΔΔCt^. Details of the primer sequences were as follows: GBE1 forward, 5′-GGAGATCGACCCGTACTTGAA-3′ and reverse, 5′-ACATCTGTGGACGCCAAATGA-3′; and HIF1*α* forward, 5′-ACCTACTGCTAATGCCACCACT-3′ and reverse, 5′-ACTCCTTTTCCTGCTCTGTTTG -3′.

### Western blot analysis

Following electrophoresis, the proteins were transferred onto polyvinylidene fluoride membranes (Millipore, Bedford, MA). The membrane was blocked in TBS-T buffer (20 mM Tris-HCl, pH 7.5; 150 mM NaCl; and 0.05% Tween-20) containing 5% (w/v) nonfat milk at room temperature (22 °C) for 1 h and then probed overnight with antibodies against GBE1 (Abcam, EP11113), FBP1 (Proteintech Group, 12842-1-AP), NF-κB (Cell Signaling Technology, D14E12), phosphorylated NF-κB (Cell Signaling Technology, 93H1), HIF-1*α* (Proteintech Group, 66730-1-Ig and Cell Signaling Technology, D2U3T) and β-actin (Cell Signaling Technology, 8H10D10) at 4 °C, followed by incubation with horseradish peroxidase-conjugated anti-IgG for 1 h at room temperature. Detection was performed with the SuperSignal West Femto maximum sensitivity substrate trial kit (Pierce, Rockford, IL, USA).

### Metabolism assays

The principle of cell bioenergy testing is based on the continuous injection of substances with different properties into the cellular oxidative phosphorylation system to emphasize the metabolic activities of the living cells. The ECARs and OCRs were detected using a glycolysis stress test kit and a Mito stress test kit^46^, respectively, on a Seahorse Extracellular Flux (XF-96) analyzer (Seahorse Bioscience, Billerica, MA, USA). Mitochondrial function was analyzed in the presence of classical modulators of the electron transport chain, including oligomycin, FCCP (carbonylcyanide p-trifluoromethoxyphenylhydrazone), and rotenone. The compounds used for glycolysis stress testing were glucose, oligomycin A, and 2-deoxy-d-glucose (2-DG; inhibits glycolysis). Normalized data for determining the number of cells were detected by YO-PRO®-1 Assay (Thermo Fisher Scientific). OCR was calculated in pmoles min^−1^ and ECAR in mpH min^−1^.

### Glycogen quantification and PAS staining

Glycogen levels were analyzed using a glycogen assay kit (BioVision Inc., Milpitas, CA, USA) according to the manufacturer’s instructions.

Glycogen was detected in the cells according to the standard PAS staining technique. Amylase (Sigma-Aldrich) was used to verify that the staining was specific for glycogen. Image acquisition was performed with an AxioVert 135 microscope (Zeiss, Jena, Germany).

### Electron microscopy

The specific staining of polysaccharides was performed using the periodic acid-thiocarbohydrazide-osmium tetroxide (PATO) method^47^. Transmission electron microscopy staining for the PATO method revealed stained electron-dense deposits in the cytoplasmic structures.

### ROS detection and NADP^+^/NADPH quantification

Intracellular ROS were analyzed using the 2′,7′-dichlorofluorescein diacetate (DCFH-DA) fluorescent probe. Cells were pretreated with carboxy-DCFH-DA (H2DCFDA; 4 μmol L^−1^; Sigma-Aldrich) and then stained with surface markers to monitor ROS levels.

The NADP^+^/NADPH ratio was detected by a NADP^+^/NADPH quantification kit (BioVision).

### Glucose uptake and lactate acid assays

Glucose uptake was analyzed according to the manufacturer’s instructions (186689-07-6; Cayman Chemical, Ann Arbor, MI, USA). Cells were collected and resuspended in glucose-free medium with 2-deoxy-2-[(7-nitro-2,1,3-benzoxadiazol-4-yl) amino]-d-glucose (2-NBDG; 100 μM). After incubation for 2 h, the cells were analyzed to detect fluorescence by flow cytometry.

The lactate acid concentration of the samples was detected using a standard curve. Cells (2 × 10^6^) were seeded evenly onto six-well plates with medium (3 mL) and cultured for 48 h, after which the cell culture medium was centrifuged and prepared for the lactate assay using a lactate assay kit (Sigma-Aldrich). Sample supernatant (50 μL) and master reaction mix (50 μL) were added to 96-well plates, and the OD was measured at 570 nm.

### Metabolite profiling

Metabolites were extracted from cell pellets and divided equally for analysis on gas chromatography/mass spectrometry (GC/MS) and liquid chromatography-tandem mass spectrometry (LC-MS/MS) platforms. Metabolite levels were normalized to that of the protein content. Five replicates per group were analyzed. Biochemical data were analyzed by Welch’s two-sample *t* test.

### IHC and immunofluorescence staining

Immunohistochemistry and immunofluorescence were performed as previously described^48^. Anti-GBE1 (1:300; Abcam), anti-Ki-67 (1:300; Abcam), anti-caspase 3 (1:300; Abcam), and anti-HIF1*α* (1:300; Proteintech, Rosemont, IL, USA) were used as primary antibodies. For immunofluorescence, Cy3- and FITC-conjugated secondary antibodies (1:500; BioLegend, San Diego, CA, USA) were used to detect primary antibodies. The samples were then imaged using a fluorescence microscope (IX71; Olympus, Japan).

### Chromatin immunoprecipitation (ChIP) assay

A ChIP assay was performed with anti-HIF1*α* (2.5 µg) and goat anti-rabbit IgG (Abcam) using the Simple ChIP^TM^ enzymatic chromatin IP kit (Cell Signaling Technology, Danvers, MA, USA) according to the manufacturer’s instructions. Subsequently, detection of the GBE1 promoter with HIF*α*-binding sites was performed by qPCR under the following conditions: 40 cycles of denaturation at 95 °C for 10 s and annealing at 58 °C for 30 s, followed by extension at 60 °C for 30 s. The following primers were used: GBE1-(-1620) forward, 5′-AGTGGCCTGCATAAGAGTGACA-3′ and reverse, 5′-AATAAAAACCCGAAGCAGGACA-3′; GBE1-(-968) forward, 5′-AATAGTTGTCCTGCTTCGGG-3′ and reverse, 5′-ATTAATCAGCCGTGGGCCTT-3′; and GBE1-(-728) forward, 5′-TCACTGTAAGTGGCAGAGTGG-3′ and reverse, 5′-TCCTAAGTTTGCCAGGTCACG-3′. The fold enrichment was normalized to the level of IgG and quantified using the 2^−ΔΔCq^ method.

### Luciferase reporter assay

A549 cells were seeded in a 24-well plate and cotransfected with pGL3-control (0.5 μg; positive control; PC), pGL3-basic (negative control; NC) or the pGL3-GBE1 promoter, and pRL-TK plasmid DNA (0.5 μg) using Lipofectamine 3000 (Invitrogen, Carlsbad, CA, USA). Then, the cells were lysed with passive lysis buffer, and reporter gene expression was assessed using a dual-luciferase reporter assay system (Promega, Madison, WI, USA) according to the manufacturer’s instructions. A luciferase activity was measured using an EnSight multimode plate reader (PerkinElmer, MA, USA).

### Cell proliferation and apoptosis assays and cell cycle analysis

Cell proliferation assays were performed using Cell Counting Kit-8 (CCK-8; Dojindo, Japan) according to the manufacturer’s instructions.

Cells were harvested and suspended in Annexin V-binding buffer to a final concentration of 1 × 10^6^ cells mL^−1^. Next, the cells were incubated with Alexa Fluor 647 Annexin V (BioLegend) for 15 min at 4 °C in the dark, after which PI (propidium iodide, Sigma-Aldrich) was added. The samples were immediately examined by flow cytometry (FACSCanto II; BD Biosciences, Franklin Lakes, NJ, USA).

A cell cycle analysis was performed using RNaseA and PI staining measured by flow cytometry. The percentage of the cells in the G0/G1, S, and G2/M phases was determined using FlowJo software (FlowJo LLC, Ashland, OR, USA).

### Migration, invasion and colony formation assays

Migration assays were performed using a Transwell system (8.0-μm pore size, 24-well insert). Matrigel (BD Biosciences) was plated onto the wells for the invasion assays. Cells (5 × 10^4^) were added to the upper chamber, and medium supplemented with FBS (600 μL) was added to the lower chamber. Then, the cells were incubated under standard culture conditions for 24 h (migration assay) or 48 h (invasion assay).

Cells (2 × 10^2^ or 5 × 10^2^ per well) were seeded onto six-well plates and incubated at 37 °C in a 5% CO_2_ humidified incubator for 10 d. At the end of the assay, the cultures were fixed in 4% paraformaldehyde for 15 min and stained with 0.1% crystal violet for 5 min.

### Sphere formation assay

Sphere formation assays were performed by seeding 5 × 10^3^ cells/well in ultra-low attachment 24-well plates (Corning Inc., Corning, NY, USA) and culturing them in DMEM/F12 (Sigma-Aldrich) containing B27 supplement (Gibco), Epidermal Growth Factor (20 ng mL^−1^; PeproTech, Rocky Hill, USA), and Fibroblast Growth Factor-basic (20 ng mL^−1^; PeproTech). After culturing for 7 d, the sphere number was determined via microscopy (Leica, Mannheim, Germany).

### Tube formation assay

Human umbilical vein endothelial cells (HUVECs) were digested and counted, after which 3 × 10^4^ cells per well were added to plates. Four hours after cell incubation at 37 °C, tube formation was observed under a microscope, and statistical analysis was performed using ImageJ software (NIH, Bethesda, MD, USA).

### IncuCyte™ cell migration and proliferation assay

We performed a monolayer scratch assay using the IncuCyte ZOOM™ live-cell imaging system (Essen BioScience, Ann Arbor, MI, USA). IncuCyte live-cell imaging enables noninvasive, fully kinetic measurements of cell growth based on area (confluence) metrics. Cells in the logarithmic growth phase were seeded onto 96-well plates at 3 × 10^4^ cells/well. When the cell fusion rate reached 90% or higher in conventional culture, the scratches were stained with a 96-well scratcher provided by IncuCyte ZOOM™. After washing the residual cells with PBS, fresh serum-free medium (200 μL) was added, and the cells were cultured in an IncuCyte ZOOM™ incubator. The results of the experiment were analyzed after the cells were in culture for 24 h.

### Global methylation analysis and methylation-specific PCR (MSP)

DNA methylation profiling (called MethylRAD) was performed as previously described^49^ using methylation-dependent restriction enzymes and Mrr-like enzymes to capture 32-bp methylated DNA fragments from the whole genome for use in high-throughput sequencing.

Modified DNA was purified with Wizard DNA purification resin (Promega). The following primers were used: methylated DNA-specific sense, 5′-TTTATAGTGCGGGTGGAGGGTAC-3′, and antisense, 5′-ACAAAATCGAAAATCCTCCCTACG-3′; and unmethylated DNA-specific sense, 5′-TTATAGTGTGGGTGGAGGGTATGG-3′, and antisense, 5′- CAAAATCAAAAATCCTCCCTACAAT-3′. PCR samples were resolved by electrophoresis using a 2% agarose gel and stained with ethidium bromide.

### Bisulfite modification for DNA methylation analysis

Genomic DNA extracted from formalin-fixed, paraffin-embedded tissues was modified by sodium bisulfate using a EpiTect bisulfite kit (Qiagen, Hilden, Germany) according to the manufacturer’s instructions. Methylation status was analyzed according to the BSP of the CpG islands. The amplified products were cloned into a pMD-18T simple vector (TaKaRa Bio, Shiga, Japan), and five independent clones were sequenced. To analyze the methylation status of the 5′ CpG islands in FBP1 in A549 cells, we also performed an MSP analysis. Published MSP primer pairs designed to specifically amplify either unmethylated or methylated FBP1 promoter-region DNA after bisulfite conversion were used^50,51^.

### Glucose metabolism RT^2^ profiler PCR array

The RT^2^ Profile PCR array for human glucose metabolism (Qiagen) was performed using the first strand cDNA synthesized as descried above. The CT values were uploaded to the data analysis portal provided by Qiagen (http://www.qiagen.com/us/shop/genes-and-pathways/dataanalysis-center-overview-page/). PCR array data were analyzed using Qiagen RT^2^ Profiler PCR data analysis software and were considered significant at >2-fold change and *P* < 0.05.

### Molecular docking

We performed a molecular docking study based on the crystal structure of GBE1 using AutoDock Vina 1.1.2; all images were generated in UCSF Chimera 1.8^51^. The protein structure of GBE1 was obtained from the Protein Data Bank (PDB, ID: 5CLT), and the PDB file was processed by removing water molecules and cations before the subsequent docking step. The active site was similar to the reported site^52–54^. The molecular docking correlation parameters were as follows: the center grid box dimensions were X center, 67.374; Y center, 9.03, and Z center, −0.328; the number of points in the X-dimension, Y-dimension, and Z-dimension were set to 16, 16, and 18, respectively; and the other chosen parameters were num_modes = 9 and exhaustiveness = 16. The lowest energy conformation was chosen for the binding model analysis.

### Xenograft nude mouse model

Six female BALB/c nude mice (Vital River Laboratory Animal Technology Co. Ltd., Beijing, China) aged 4–6 weeks and weighing 16–20 g were randomly divided into two groups (three mice/group). To establish the lung cancer xenograft model, both groups received hypodermic injections of either scrambled shNC or shGBE1 A549 cells (1 × 10^7^ cells in 100 μL PBS). In the flavopiridol experiment, 3 d after the cell injection, the mice were randomly divided into two groups and treated with flavopiridol, or the DMSO control for 41 d. Flavopiridol was administered three times per week (8 mg/kg). Mice were inspected every 3 d, and tumor volumes were calculated using the following formula: (length × width^2^)/2. The mice were sacrificed by cervical dislocation 31 d after cell implantation. The tumors were collected for protein and RNA extraction and IHC analysis.

For the detection of bioluminescence, the mice were anesthetized with 4% (v/v) chloral hydrate (Sigma-Aldrich) and intraperitoneally injected with 150 mg/kg luciferin (Promega). The bioluminescence signals were quantified after biodistribution in vivo using a IVIS SPECTRUM/CT noninvasive optical imaging system (PerkinElmer). For luciferase quantification, data are expressed as the average radiance (photons/second/cm^2^/steradian).

### Statistical analysis

SPSS 19.0 software (IBM, NY, USA) or Prism 6 (GraphPad Software Inc., La Jolla, CA, USA) was used to analyze the data. An independent sample or paired *t* test was performed to analyze the differences between two groups with normally distributed continuous variables. Pearson’s coefficient correlation or linear regression analysis was used to analyze the relationship between specific gene expression levels. The Kaplan−Meier method was used to establish survival curves, and the log-rank test was used to compare survival differences. In all cases, a two-tailed *P* value of <0.05 was considered significant.

## Supplementary information

Supplementary Materia 1
