# Supplementary material for: Hypoxia-induced GBE1 expression promotes tumor progression through metabolic reprogramming in lung adenocarcinoma
Source: Signal Transduct Target Ther. 2020 May 22;5:54. doi: 10.1038/s41392-020-0152-8 (PMC7242448; doi:10.1038/s41392-020-0152-8)
Supplement: Supplementary file 1 — Supplementary Materia 1 [file 41392_2020_152_MOESM1_ESM.docx]

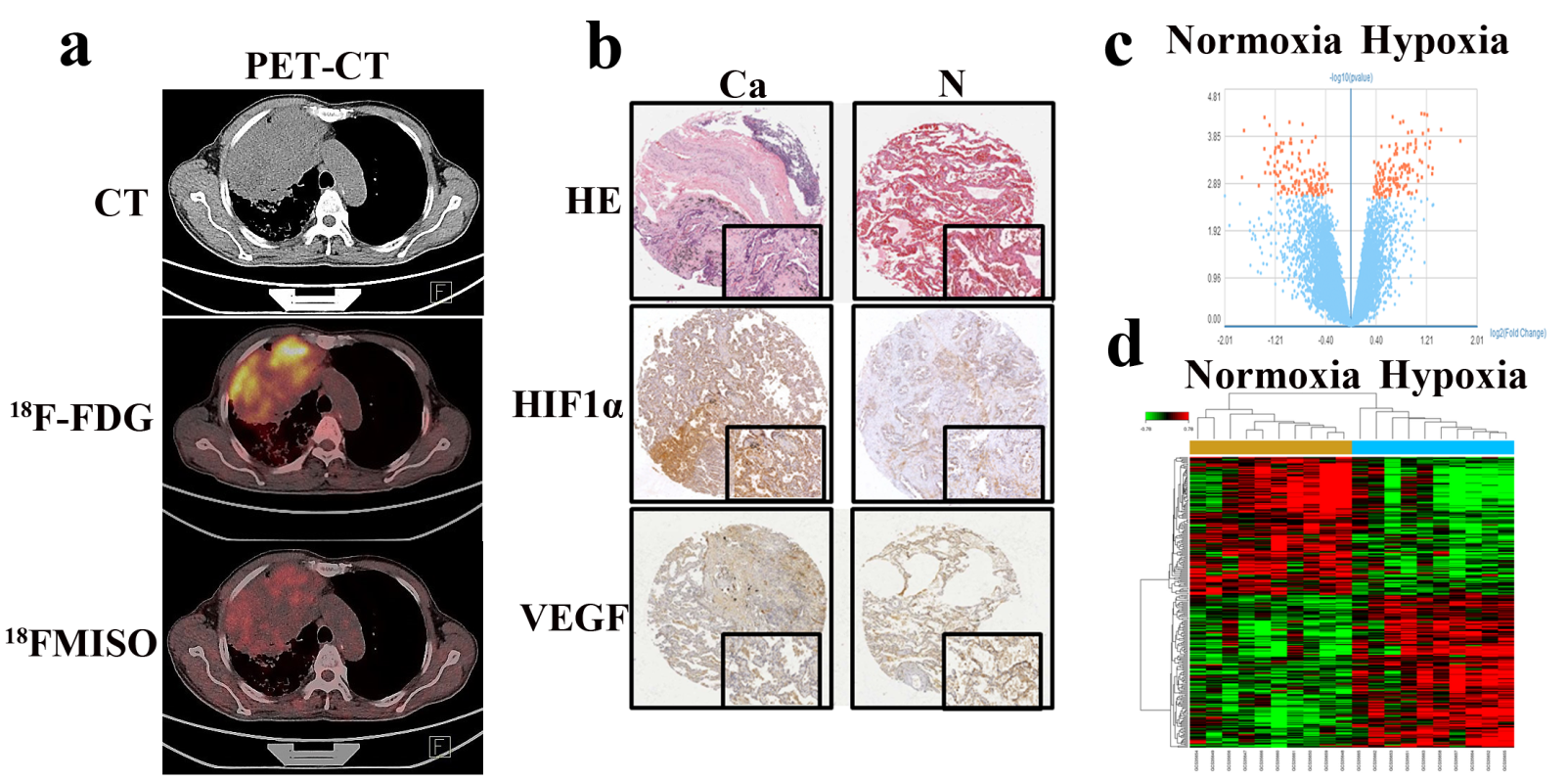


**Fig. S1** **Detection of hypoxia in LUAD tissues.** (**a**) Fusion image of PET/CT of one LUAD patient, including ^18^F-FDG and ^18^FMISO PET. (**b**) The expression of hypoxia-relevant genes (HIF1α and VEGF) in serial sections derived from LUAD and peritumor tissues. (**c, d**) Graphic display of gene expression profiling in gene network analysis (**c**) and heatmap format (**d**) from hypoxic and normoxic sections of LUAD.


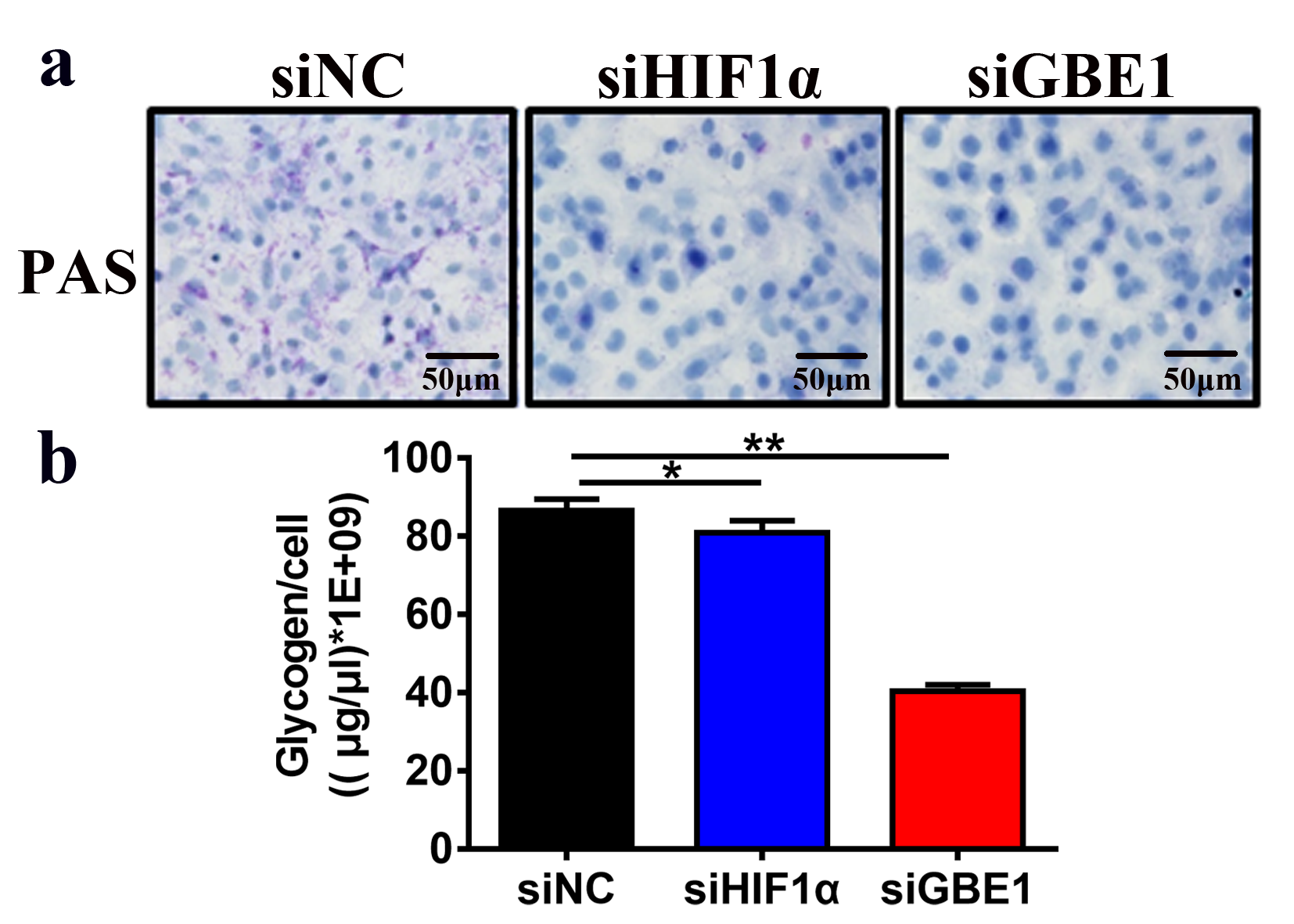


**Fig. S2 Glycogen levels analyzed by PAS staining.** (**a**) PAS staining of glycogen in siHIF1α and siGBE1 A549 cells. One representative micrograph is shown (magnification, 200×). (**b**) A histogram of the results from (**a**) is presented. Data are represented as means ± SD. **P* < 0.05, ***P* < 0.01.


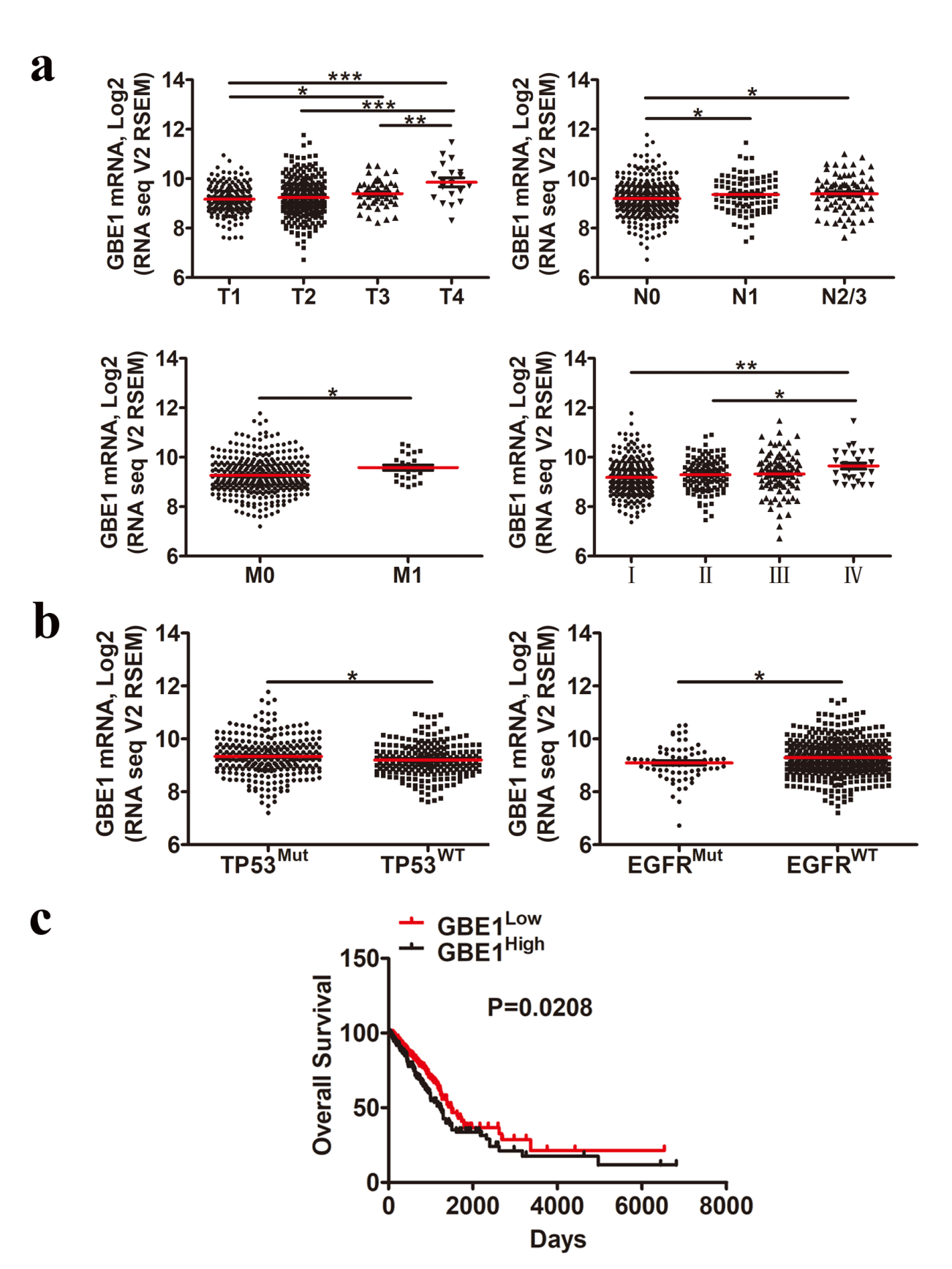


**Fig. S3 Correlation between GBE1 expression and clinical pathological parameters from TCGA dataset.** (**a**) GBE1 expression during different stages (T1–T4, N0–N2/3, M0–M1, and I–IV) of LUAD patients retrieved from TCGA dataset. (**b**) Correlation between TP53 and EGFR mutation with GBE1 expression. (**c**) Kaplan-Meier curves show the association between GBE1 expression and OS. Data are represented as means ± SD. **P* < 0.05, ***P* < 0.01, ****P* < 0.001.


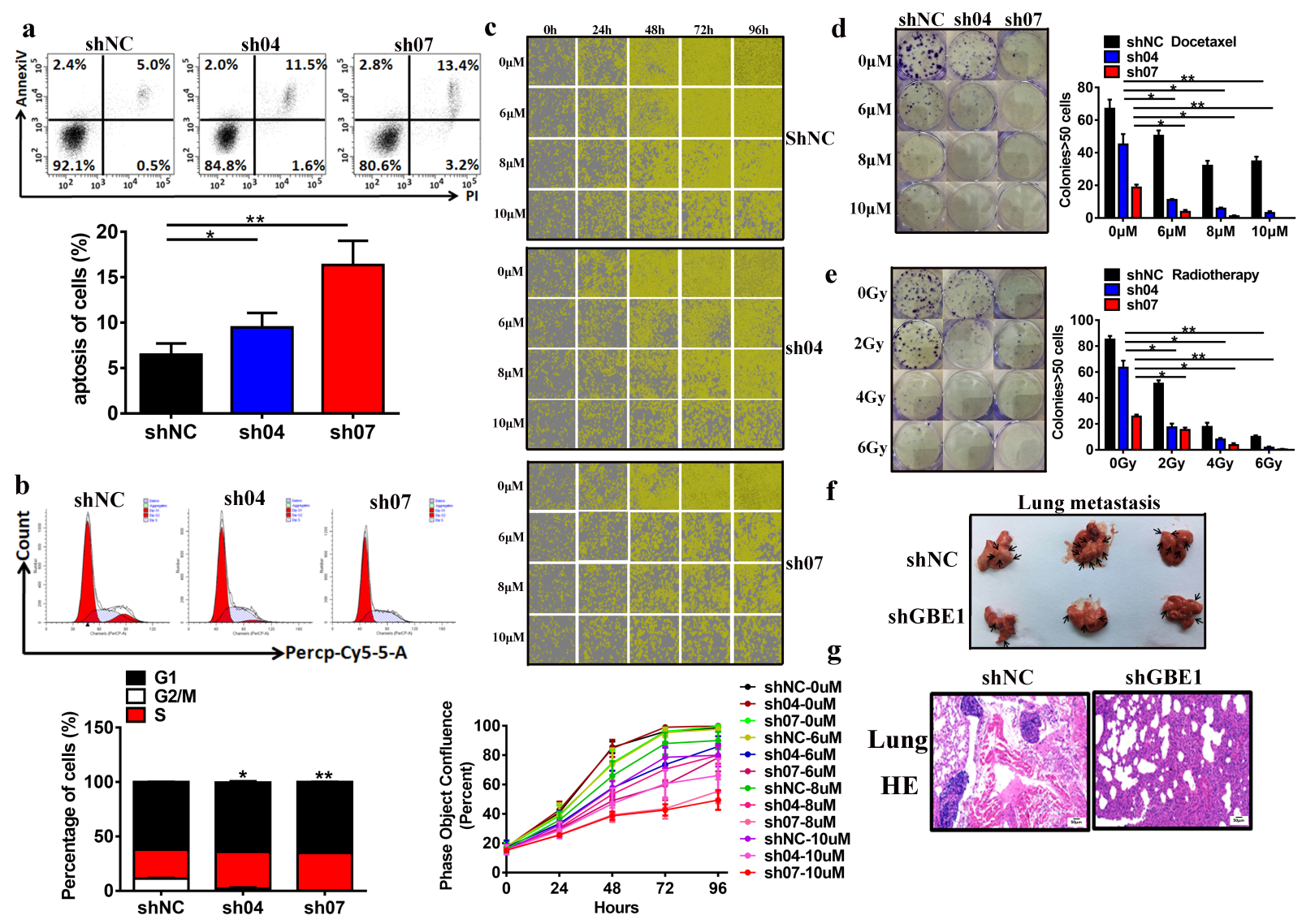


**Fig. S4 The effect of GBE1 knockdown on tumor progression in vitro and in vivo.** (**a, b**) A549 apoptosis analyzed by flow cytometry (**a**) and cell cycle by flow cytometry (**b**). (**c**) Cell proliferation of shGBE1 A549 cells before and after treatment with docetaxel (0, 6, 8, and 10 µM) was analyzed by IncuCyte ZOOM™ assays. (d, e) Cell clone formation ability of shGBE1 A549 cells before and after treatment with docetaxel (0, 6, 8, and 10 µM) (**d**) and radiotherapy (0, 2, 4, and 6 Gy) (**e**). (**f**) Metastasis to lungs of shGBE1 and shNC A549 cells. (**g**) HE staining of shGBE1 and shNC A549 cell lung metastasis. Data are represented as means ± SD. **P* < 0.05, ***P* < 0.01.


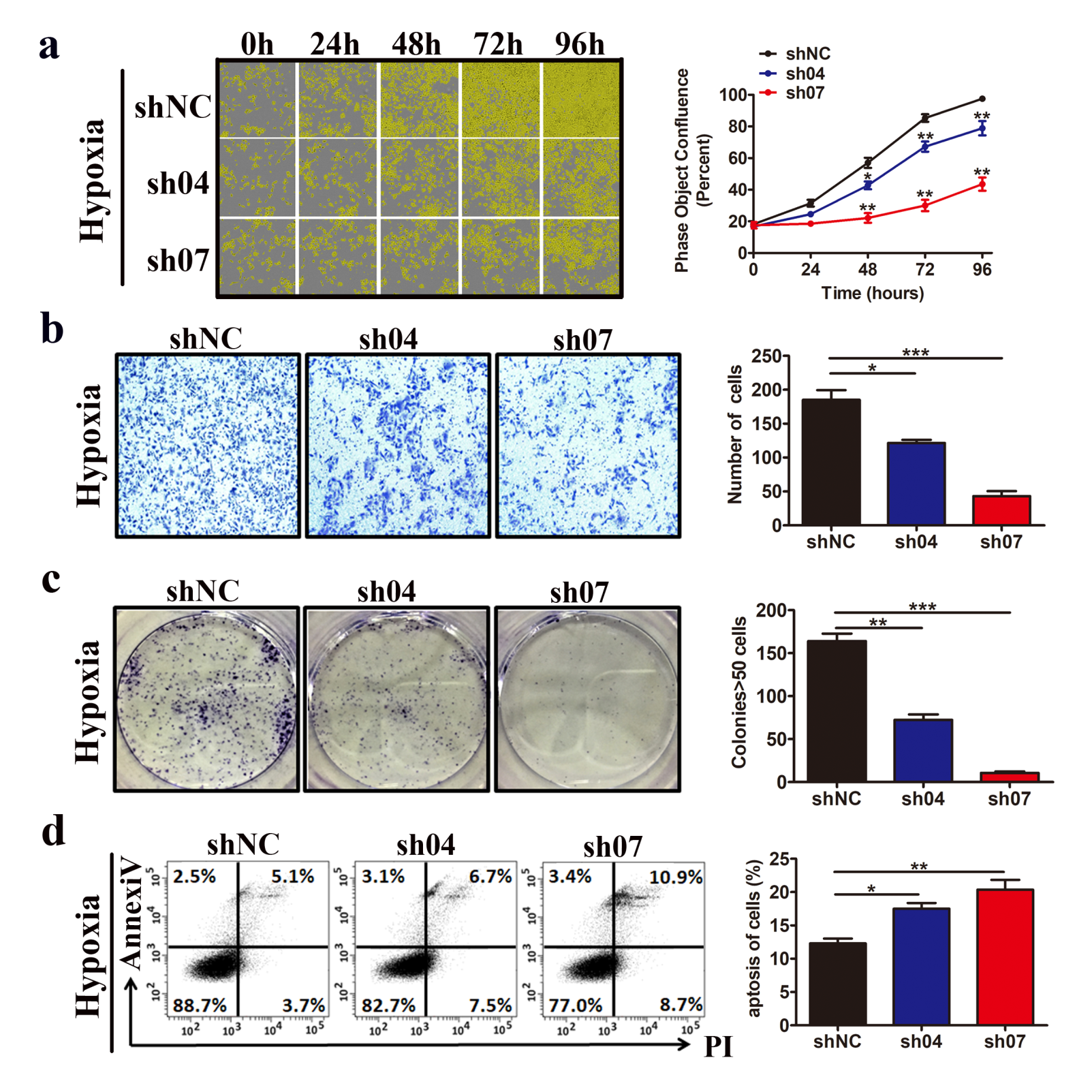


**Fig. S5** **The effect of GBE1 knockdown in A549 cells under hypoxia on tumor progression in vitro.**

(**a**) Cell proliferation of shGBE1 A549 cells under hypoxia was analyzed by IncuCyte ZOOM™ assays. (**b**) Cell migration and invasion of shGBE1 A549 cells under hypoxia was analyzed by Transwell assays. (**c**) Cell clone formation ability of shGBE1 A549 cells under hypoxia. (**d**) A549 cell apoptosis under hypoxia was analyzed by flow cytometry. Data are represented as means ± SD. **P* < 0.05, ***P* < 0.01, ****P* < 0.001.


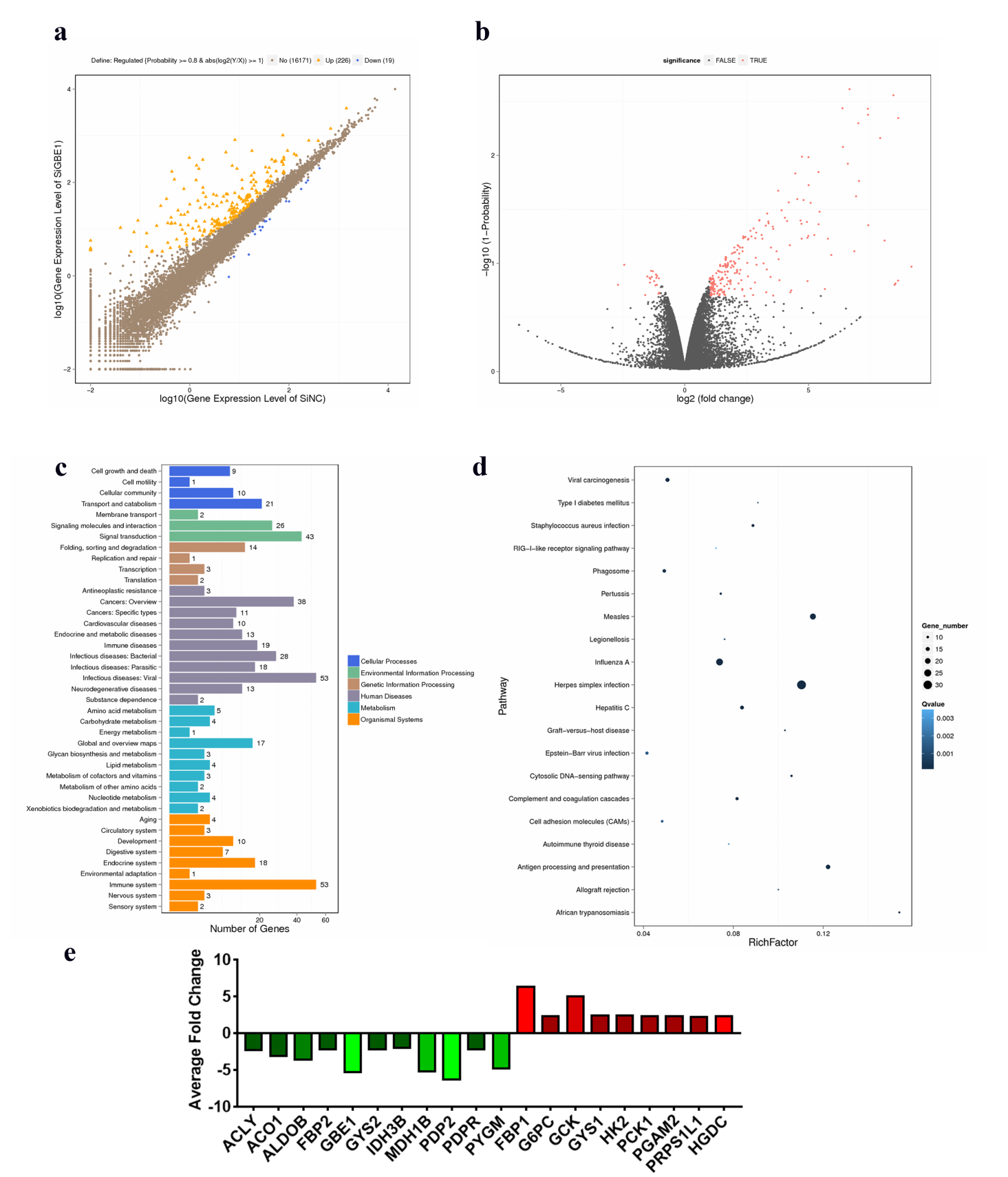


**Fig. S6** **Analysis of differentially expressed genes (DEGs) induced by GBE1 knockdown.** (**a, b**) Scatter plot (**a**) and volcano plot (**b**) of global gene expression pattern in shGBE1 and control A549 cells. Each dot is a DEG. Down- (blue dots) and upregulated (orange dots) genes are presented as DEGs. (**c, d**) GO functional classification (**c**) and KEGG pathway analysis (**d**) showing the top ranking pathways associated with GBE1; 20 of the most enriched pathways (with lowest Q value) are presented. (**d**) Changes in metabolic genes after GBE1 knockdown was analyzed by glucose metabolism PCR array. Genes showing > 2-fold change are included.


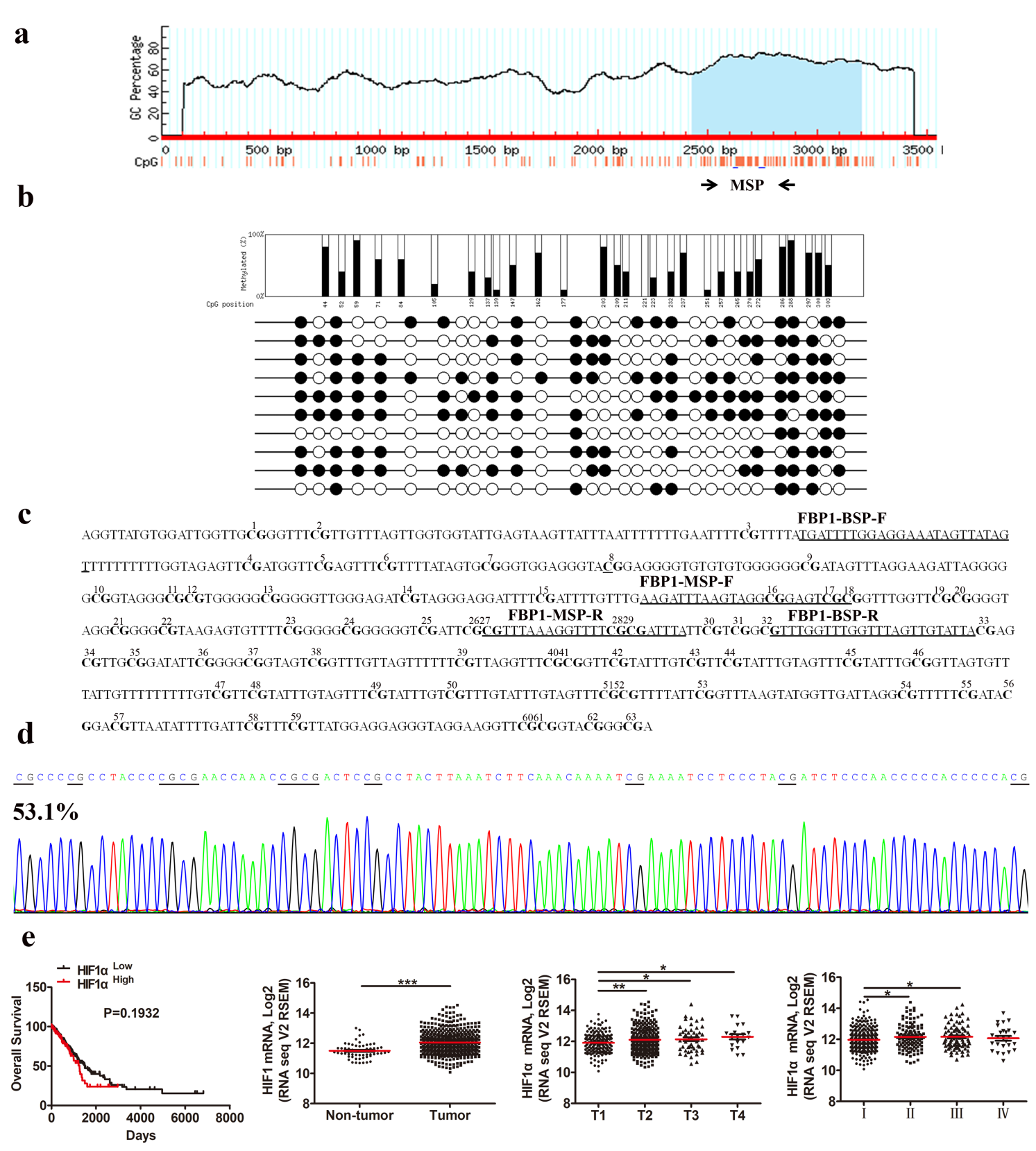


**Fig. S7 FBP1 expression in GBE1-knockdown cells was correlated with high methylation levels.** (**a**) One typical CGI was found around FBP1 exon 1. MSP primers are indicated by the arrows. (**b**) FBP1 CGI methylation was analyzed by BSP, where each circle represents a CpG site and the black circle represents a methylated CpG site. A line of circles represents an individual clone. (**c**) The FBP1 promoter region, which was numbered 1 to 63 by CpG dinucleotides, and primers for BSP and MSP are underlined. (**d**) Methylation of 63 CpG sites in the promoter region of FBP1 in A549 cells. Sequencing results showing that FBP1 expression in A549 cells was silenced and that densely methylated CpG islands were found. (**e**) Correlation between HIF1α expression and clinical pathological parameters from TCGA dataset. Data are represented as means ± SD. **P* < 0.05, ***P* < 0.01, ****P* < 0.001.


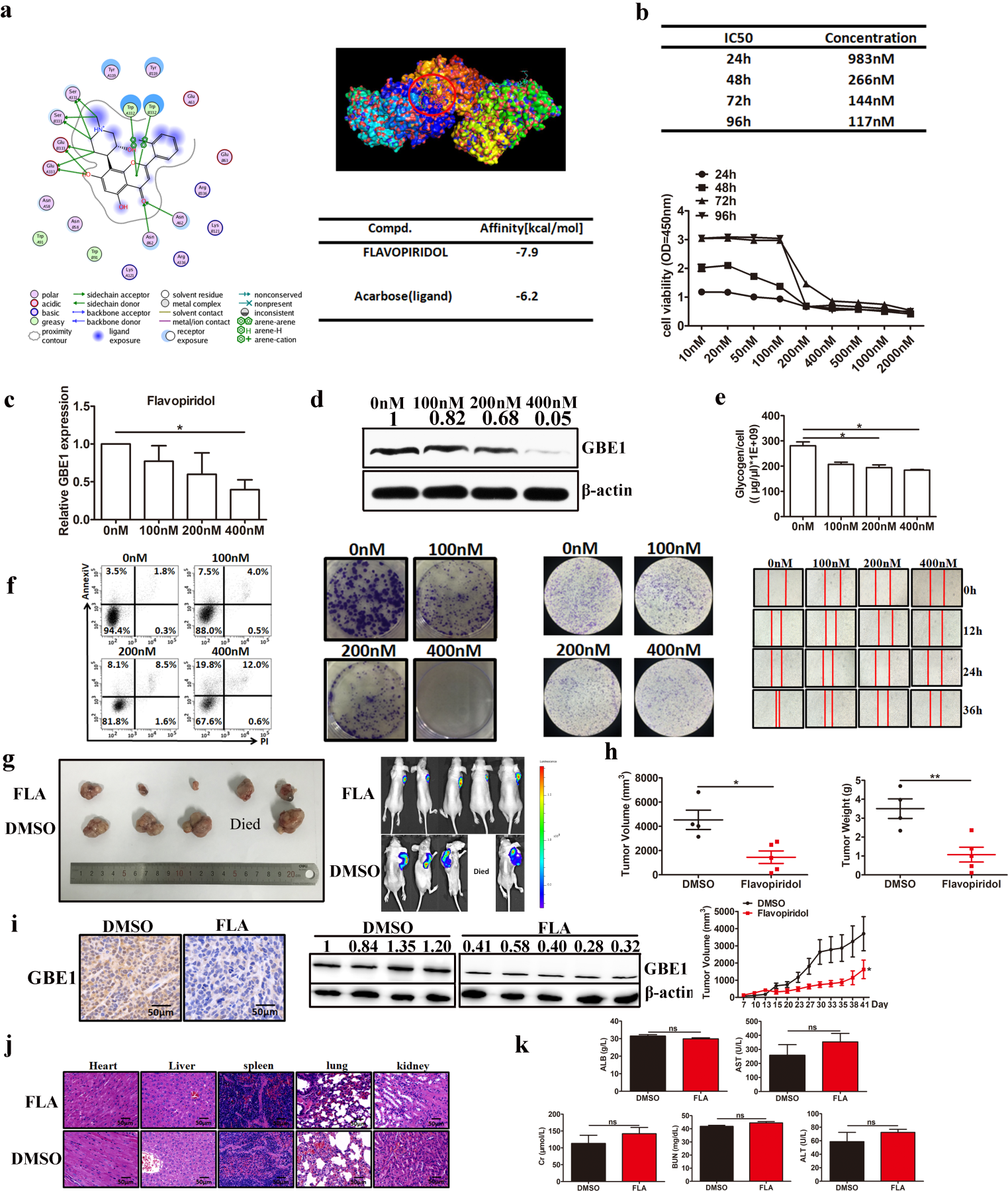


**Fig. S8 Flavopiridol as a potential inhibitor of GBE1 for LUAD treatment.** (**a**) Schematic representation of the molecular docking study of flavopiridol based on the crystal structure of GBE1 (PDB code: 5CLT) in complex with acarbose. (**b**) Absolute IC_50_ values of flavopiridol were determined by CCK-8 assay. (**c**) qPCR and (**d**) western blotting analysis of GBE1 expression. (**e**) Glycogen content measurement before and after treatment with flavopiridol (0, 100, 200, and 400 nM). (**f**) The effect of flavopiridol (0, 100, 200, and 400 nM) on cell apoptosis, colony formation, cell migration, and invasion. (**g**) Tumor growth was measured using a caliper and an in vivo imaging system after treatment with flavopiridol. (**h**) Tumor volumes were measured at the indicated time points after cell implantation (once per 3 d), and tumor weight and volume of xenografts were measured at the last time point after cell implantation. (**i**) GBE1 expression in treated xenografts was analyzed by IHC and western blotting. (**j**) Representative HE staining of heart, liver, spleen, lung, and kidney lesions that were treated with flavopiridol. (**k**) Concentration of albumin (ALB), aspartate aminotransferase (AST), alanine aminotransferase (ALT), creatinine (Cr), and blood urea nitrogen (BUN), in sera of mice treated with flavopiridol. Data are represented as means ± SD. **P* < 0.05, ***P* < 0.01.


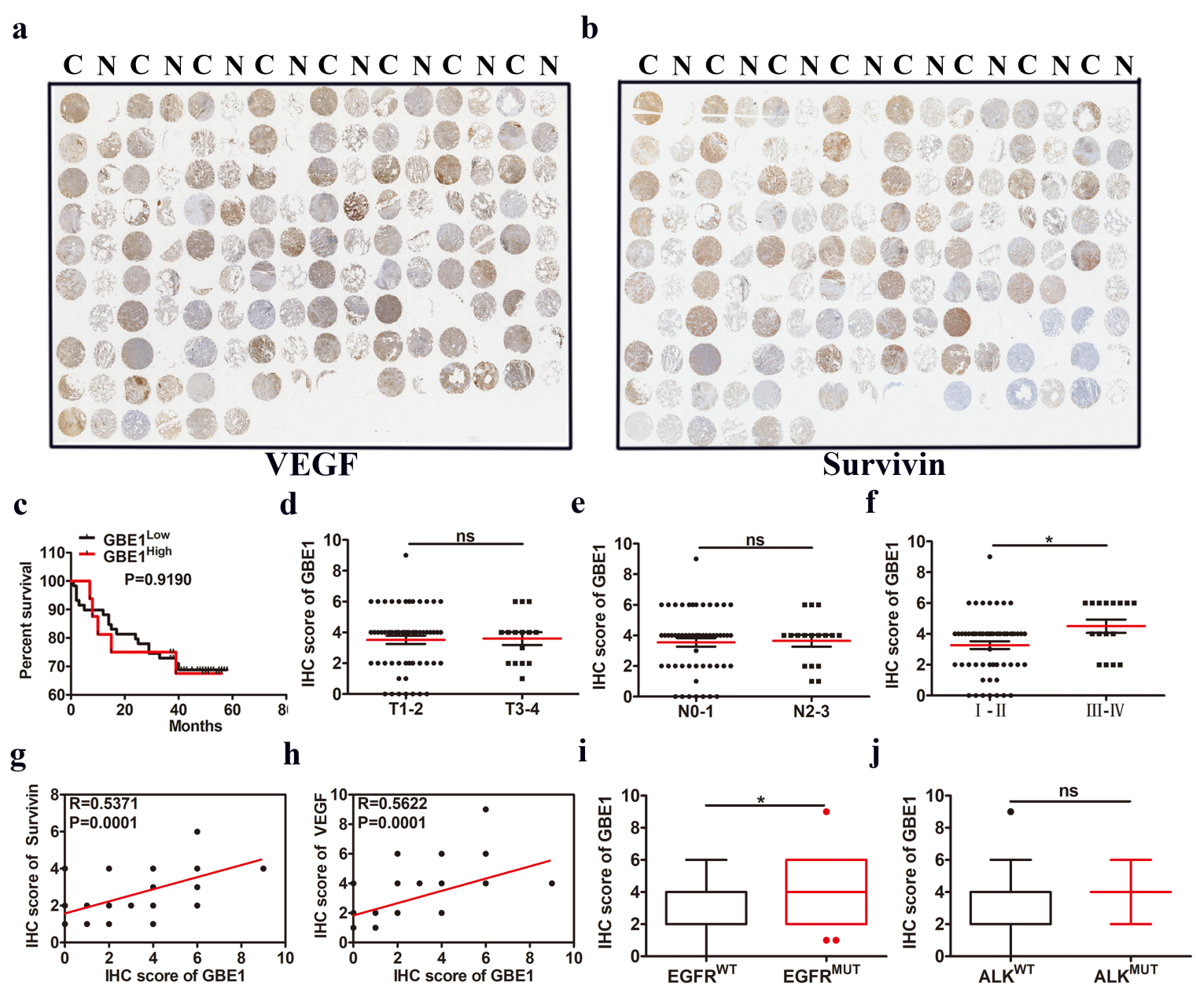


**Fig. S9 Correlation between gene expression and clinical pathological parameters.** (**a, b**) IHC staining of survivin (**a**) and VEGF (**b**) expression from a representative human LUAD tissue microarray. (**c**) Kaplan-Meier curve showing the association between GBE1 expression in normal lung tissues and OS. (**d–f**) GBE1 expression in adjacent normal tissues of LUAD patients during different stages (T1–T4, N0–N2/3, M0–M1, and I–IV). (**g, h**) Correlation between GBE1 expression and survivin (**g**) and VEGF (**h**) expression in LUAD tissues using IHC results. (**i, j**) Comparison between GBE1 levels in LUAD tissues with or without EGFR (**i**) and ALK (**j**) mutations using IHC results. Data are represented as means ± SD. **P* < 0.05.
